# Investigation of Drought Stress on Chickpea (*Cicer arietinum* L.) Genotypes Employing Various Physiological Enzymatic and Non-Enzymatic Biochemical Parameters

**DOI:** 10.3390/plants13192746

**Published:** 2024-09-30

**Authors:** Ruchi Asati, Manoj Kumar Tripathi, Rakesh Kumar Yadav, Niraj Tripathi, Ravendra Singh Sikarwar, Prakash Narayan Tiwari

**Affiliations:** 1 Department of Genetics & Plant Breeding, College of Agriculture, Rajmata Vijayaraje Scindia Krishi Vishwa Vidyalaya, Gwalior 474002, India; ruchiasati.95@gmail.com (R.A.); rakeshyadav07081996@gmail.com (R.K.Y.); ravendra484@gmail.com (R.S.S.); 2 Department of Plant Molecular Biology and Biotechnology, College of Agriculture, Rajmata Vijayaraje Scindia Krishi Vishwa Vidyalaya, Gwalior 474002, India; tiwarisprakashn051194@gmail.com; 3 Directorate of Research Services, Jawaharlal Nehru Krishi Vishwa Vidyalaya, Jabalpur 482004, India

**Keywords:** drought, ascorbate peroxidase, catalase, chlorophyll content, RWC, MSI, SWD, CTD, superoxide dismutase

## Abstract

Drought stress is a universal crisis in sustaining the growth and production of major legumes, including the chickpea. Drought severely reduces the biomass of chickpea plants, with the effect on leaves appearing the most apparent. The aim of this study was to investigate, using various physiological and biochemical markers throughout the pod filling stage, how 78 desi chickpea genotypes tolerated drought stress. Most of the evaluated characteristics showed significant variations between control and drought treatments. The mean performance of most of the investigated parameters significantly decreased under moisture-stressed conditions. RWC, SWD, MSI, and CTD were investigated under terminal drought-stressed conditions. Except for saturated water deficit (SWD), all remaining characteristics declined with increasing stress. Genotypes SAGL152210, SAGL152252, SAGL152347, SAGL22-115, and JG11 were recognized as drought-tolerant based on physiological characteristics. Biochemical markers viz., protein content, total soluble sugar, lipid peroxidation, and proline content, had an impact on osmotic adjustment. Based on non-enzymatic biochemical traits, genotypes SAGL22-115, ICC4958, ICCV201108, ICCV201107, SAGL152252, and JG11 were identified for their capability to survive under drought-stressed conditions. H_2_O_2_ content, CAT, SOD, POD, APX, and DPPH were considered antioxidant agents. Genotypes SAGL152208, SAGL22-105, SAGL22-112, ICC201108, SAGL152278, SAGL152252, SAGL162371, SAGL162390, ICC 4958, and JG315 may be considered drought-tolerant based on antioxidant activities. These genotypes are believed to be better equipped with physio-biochemical mechanisms and antioxidant defense systems at the cellular level and can be used in breeding programs to breed drought-tolerant cultivar(s). They can also be screened in the future, allowing the line(s) that have remained consistent over time to be recognized and registered as drought-tolerant donors.

## 1. Introduction

Drought pressure is still one of the most important abiotic stresses impacting plant development and causing serious yield challenges in the chickpea across the world [1,2]. It is becoming more frequent and intense as resources are depleted and the environment changes year by year. Unexpected alterations in climatic circumstances ensuing high global temperatures (heat stress) and unanticipated rainfall situations (floods and drought) are becoming important agricultural production problems [2]. Because of this, feeding the world’s expanding population is a problem for global agriculture [3]. About one-third of the world’s population currently lives in water-scarce areas, and it is believed that due to climate change and rising CO_2_ levels in the atmosphere, the intensity, duration, and frequency of drought stress will all rise. The flexibility of the legume crops in the present predominant weather edges could be the innovative adaptation in more severe climatic conditions [4].

The oldest and most widely used legume is the chickpea [5], which is also the most frequently grown crop in the world, with over fifty countries producing it [6]. The main cultivating nations of chickpeas are India, Pakistan, Turkey, Australia, and Myanmar. India contributes to most of the worldwide production (70%) and is the greatest producer [7,8]. Its cultivation now covers 15.004 million hectares (m ha), with a productivity of 1057.8 kg h^−1^ and a global production of 15.87 mt per year. According to FAOSTAT [9], India accounts for 73.78% (10.943 m ha) of the world’s total chickpea area and 73.45% (11.91 m tons) of its production [10]. Madhya Pradesh contributes more than 40% of the national chickpea production [11]. In semi-arid areas, it is mostly cultivated on marginal fields. Due to limited and irregular rainfall in these regions, chickpeas are continually exposed to severe drought and high temperatures during the flowering and maturity stages. Accordingly, there are two types of droughts that affect chickpeas: terminal droughts, where soil moisture content constantly decreases as the growing season comes to an end, and intermittent droughts, where soil moisture depends on precipitation, but rainfall is erratic and insufficient [12]. Plants are stressed by intermittent and terminal drought conditions during the vegetative and reproductive growth phases. A recent study states that up to 50% of chickpea output losses can be attributed to drought stress [13].

Diverse biochemical and physiological processes in crop plants are impacted by drought. Those changes result in a decrease in growth, a drop in chlorophyll levels, a reduction in ascorbic acid, an increase in proline accumulation, and a rise in hydrogen peroxide [14,15]. An assessment of genotypes against drought tolerance may be accomplished by employing these characteristics. Different reactive oxygen species (ROS) accumulate in plants during drought stress. These ROS induce oxidative damage to tissues and cells, which leads to cell death. In essence, plant components are harmed by the interaction between free radicals and electrons obtained from other molecules because they have a detrimental impact on the enzyme system. Under drought conditions, oxidative damage in plants is controlled by enzymatic and non-enzymatic antioxidant machineries. Superoxide, hydroxyl, hydrogen peroxide, and alkoxy radicals can alter regular cellular metabolism by oxidatively damaging proteins, membranes, and nucleic acids [16]. The endogenous defense system of the chickpea, which is composed of enzymatic and non-enzymatic antioxidants, protects it against oxidative stress at the cell level via adaptation [17]. Physiological traits such as relative water content, MSI, chlorophyll content, and leaf area index help to understand yield variation under stressed conditions. These physiological and biochemical parameters are good indicators for selecting desired genotypes against drought. These endogenous defense mechanisms detect changes in crop plants in controlled and stressful environments. The lack of adequate selection indicators, mostly morphological and physiological response, limits breeding for drought tolerance in chickpea. Research on several shoot-related factors, such as biomass, stomatal conductance, canopy temperature, and pods/plant, is still primarily focused on boosting chickpea genetic productivity under drought stress [18]. Likewise, the chickpea biochemically develops several kinds of biochemicals and antioxidants that help to mitigate the problems of reactive-oxygen-species-derived toxicity during drought stress [19,20,21]. Therefore, there is still a need for more knowledge of the genetic principles governing the different factors. Breeders may be able to develop effective breeding techniques that support the development of varieties with drought tolerance on good genetic bases by understanding the genetic makeup of such characteristics. Thus, to fill this gap, the present study was designed to evaluate the effect of terminal drought stress in chickpea genotypes by assessing the main drought-tolerant parameters. The selection of high-yielding drought-tolerant chickpea genotypes, particularly those cultivated in India, was the other objective of the study.

## 2. Results

### 2.1. Effect of Terminal Drought Stress on Physiological Traits

The study of physiological parameters includes measurements of the relative water content (RWC), saturation water deficit (SWD), canopy temperature depression (CTD), and membrane stability index (MSI). Except for the SWD, all physiological parameters were significantly reduced in all 78 chickpea genotypes under stressed conditions compared to normal conditions, and a significant difference was found among the genotypes. Only the SWD was significantly increased in all 78 chickpea genotypes under stressed conditions compared to normal conditions.

#### 2.1.1. RWC

**Figure 1:**
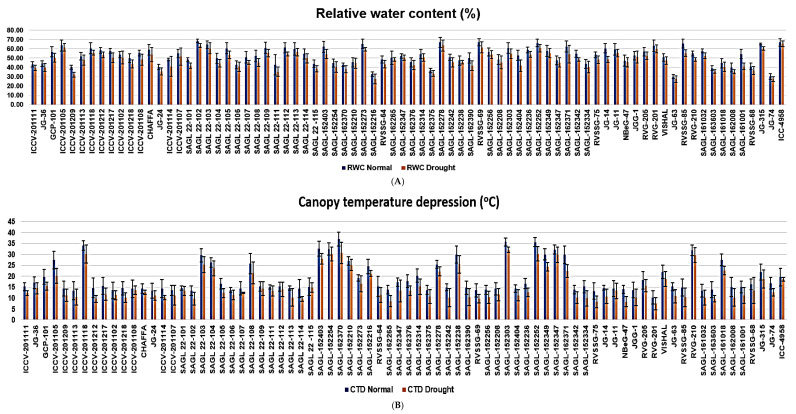

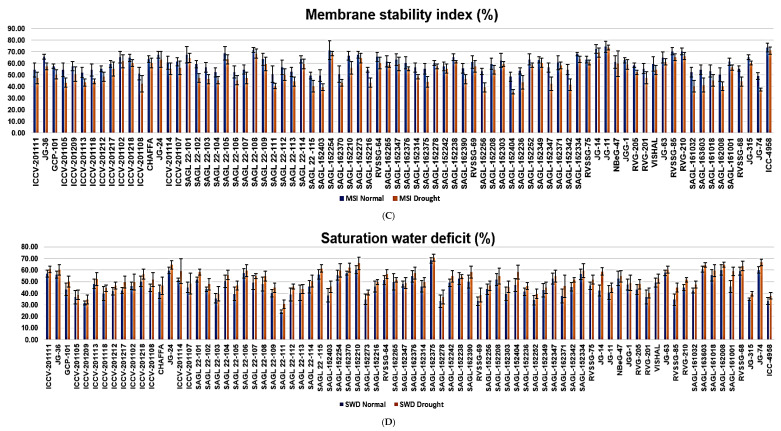
(**A**) Relative water content, (**B**) canopy temperature depression, (**C**) saturated water deficit, and (**D**) membrane stability index of 78 chickpea genotypes under control, and drought-stressed (Drought) conditions.

Under normal conditions, RWC ranged from 30.12% to 68.32%, with an average of 52.55%, whereas under stressed conditions, it ranged from 27.35% to 63.44%, with a mean of 47.61%. Under normal conditions, a higher RWC was detected in genotype SAGL22-102 (68.32%), whilst a lower value was evident in genotype JG-63 (30.12%). Likewise, a higher RWC was maintained in genotype SAGL152278 (63.44%) under stressed conditions, whereas a lower RWC was displayed by genotype JG-63 (27.35%). The minimum reduction in RWC was detected in genotype SAGL152210 (0.91%); however, the maximum decrease in RWC was observed in genotype SAGL161001 (13.33%) under stressed conditions over normal conditions (Table 1 and Figure 1A). In this investigation, under stressed conditions, the average reduction in RWC was 4.94 compared to normal conditions.

#### 2.1.2. SWD

SWD ranged from 23.98% to 63.41%, with an average of 33.29% under normal conditions, whereas under stressed conditions, it varied between 30.58% and 70.68%, with a mean 37.79%. Under normal conditions, a lower STD was measured in genotype SAGL22-111 (23.98%), while a higher value was noticed in genotype SAGL162375 (63.41%). Correspondingly, a lower STD was maintained in genotype SAGL22-111 (30.58%) under stressed conditions, whereas a higher STD was recorded in genotype SAGL162375 (70.68%). The minimum increase in STD was evident in genotype SAGL152347 (0.97%), while the maximum increase in STD was recorded in genotype JG-14 (16.53%) under stressed conditions over normal conditions. In this investigation, under stressed conditions, the average increase in STD was 5.02% compared to normal conditions (Table 1 and Figure 1B).

#### 2.1.3. CTD

Canopy temperature depression ranged from 10.32 °C to 36.89 °C, with an average of 18.92 °C under normal conditions, whereas under stressed conditions, it ranged from 7.43 °C to 32.12 °C, with a mean of 15.39 °C. Under normal conditions, a higher CTD was observed in genotype SAGL162370 (36.89 °C), while a lower value was recorded in genotype RVG201 (10.32 °C). Likewise, a higher CTD was maintained in genotype SAGL152303 (32.12 °C) under stressed conditions, whereas a lower CTD was recorded in genotype RVG201 (7.43 °C). The minimum reduction in CTD was observed in genotype SAGL 22-115 (0.42), whereas the maximum reduction was noticed in genotype SAGL162371 (7.50) under stressed conditions over normal conditions. Overall, under stressed conditions, the average reduction in CTD was 3.59 °C compared to normal conditions (Table 1 and Figure 1C).

#### 2.1.4. MSI

Membrane stability index varied between 48.37% and 74.20%, with a mean of 59.63% under normal conditions, whereas under stressed conditions, it ranged from 35.30% to 73.65%, with an average of 53.05%. In respect to genotypes, under normal conditions, higher MSI in percentage was evidenced in genotype JG11 (74.20%), while the lowest value was recorded in genotype SAGL152404 (48.37%). Higher MSI displayed by genotype JG11 (73.65%) under stressed conditions, while lower MSI was recorded in genotype SAGL152404 (35.30%). The minimum reduction in MSI was observed in genotype JG11 (0.55%), and the maximum reduction in MSI was recorded in genotype SAGL162389 (14.10%) under stressed conditions over normal conditions. Under stressed conditions, the mean reduction in MSI was 6.57% compared to normal conditions (Table 1 and Figure 1D).

### 2.2. Effect of Terminal Drought Stress on Biochemical Traits

#### 2.2.1. Non-Enzymatic Antioxidants

##### Chlorophyll a (Chl_a_)

**Figure 2:**
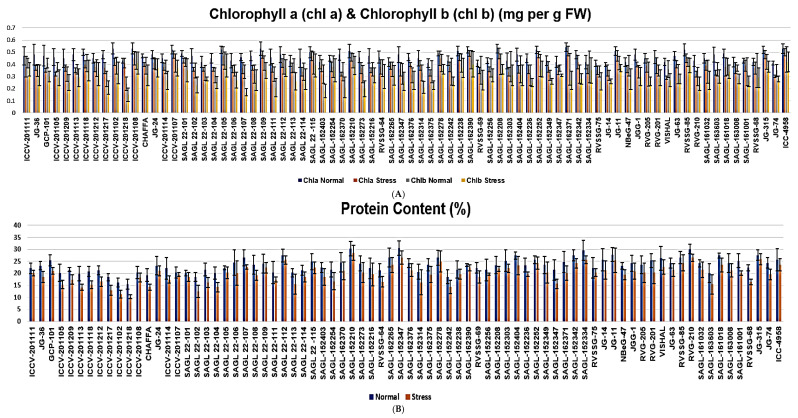

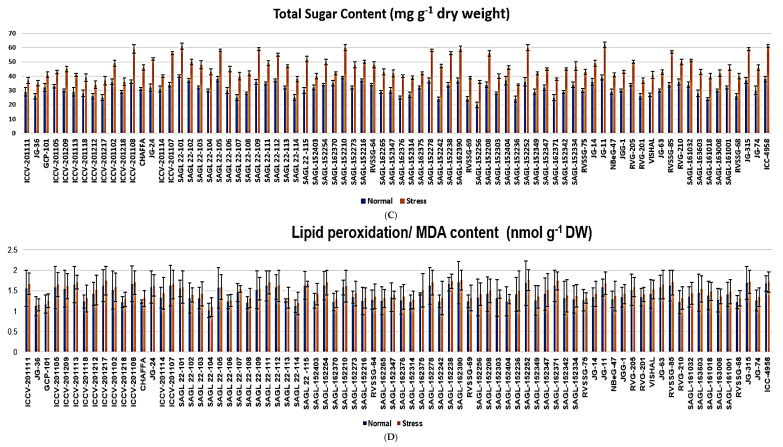

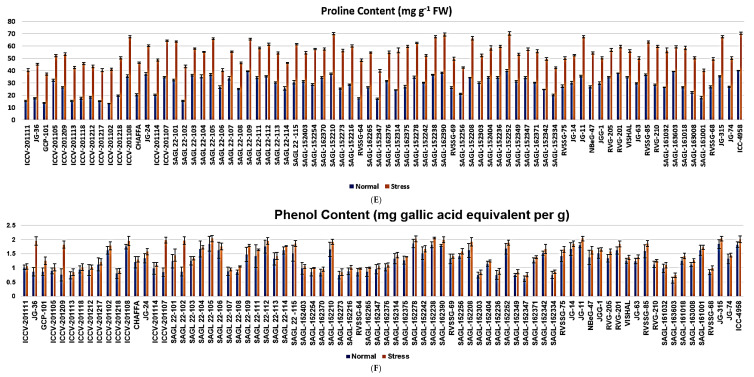
Consequence of terminal drought stress on chickpea genotypes. (**A**) Chlorophyll a and b, (**B**) protein, (**C**) sugar, (**D**) MDA, (**E**) proline, and (**F**) phenol content.

Chlorophyll a was significantly reduced in all 78 chickpea genotypes under stressed conditions compared to normal conditions, and a significant difference was found among the genotypes. Under normal conditions, it ranged from 0.4 (JG14) to 0.54 (SAGL162371) mg per g FW, with an average of 0.46 mg g^−1^ FW, whereas under stressed conditions, it ranged from 0.29 (JG63) to 0.51 (SAGL162371) mg per g FW with an average of 0.39 mg per g FW. Drought stress (normally sown) significantly decreased leaf chlorophyll a in all genotypes (0.03–0.15-fold), especially in the drought-sensitive (SAGL162370, 0.16-fold; SAGL163603, 0.15-fold) and drought-tolerant genotypes (ICCV201218, 0.02-fold), followed by genotypes SAGL22-115, SAGL152403, SAGL162265, and ICCV4958, with decreases of 0.03-fold. In this investigation, under stressed conditions, the average reduction in chlorophyll a was 0.074-fold compared to normal conditions (Table 2 and Figure 2A).

##### Chlorophyll b (Chl_b_)

Chlorophyll b was also significantly reduced in all 78 chickpea genotypes under stressed conditions compared to normal conditions, and a significant difference was evidenced among the genotypes. Under normal conditions, it ranged from 0.44 (ICCV4958 and SAGL162390) to 0.24 (SAGL 201108) mg per g FW, with an average of 0.36 mg per g FW, whereas under stressed conditions, it ranged from 0.41 (SAGL201108) to 0.15 (SAGL201218) mg per g FW, with an average of 0.29 mg per g FW. Drought stress (normally sown) significantly decreased leaf chlorophyll b in all genotypes (0.02–0.17-fold), especially in the drought-sensitive genotype SAGL22-107 (0.17-fold) and the drought-tolerant genotypes ICCV 201108, SAGL 22-115, and ICCV 4958 (0.02-fold). The average reduction in chlorophyll b under stressed conditions was 0.65-fold compared to normal conditions (Table 2 and Figure 2A).

##### Protein Content

Protein was significantly reduced in all 78 chickpea genotypes under stressed conditions compared to normal conditions, and a significant difference was observed among the genotypes. Under normal conditions, it varied from 15.4% (ICCV 201218) to 30.4% (SAGL152347), with a mean of 23.36%, whereas under stressed conditions, it ranged from 10.24% (SAGL201218) to 28.45% (SAGL152210), with an average of 19.79%. The minimum reduction in protein was observed in genotype ICCV201107 (0.78-fold), SAGL 22-115, and ICCV4958 (2-fold), whilst the maximum reduction in protein was recorded in genotype SAGL22107 under stressed conditions over normal conditions (Table 3). Under stressed conditions, the average reduction in protein was 6.55-fold compared to normal conditions (Table 3 and Figure 2B).

##### Total Sugar Content

Significant variation was recorded in total soluble sugars estimated in dry chickpea leaves. The maximum sugar content was observed in genotype SAGL22-101 (40 mg g^−1^ dry weight) and the minimum in SAGL152256 (20 mg g^−1^ dry weight) under normal conditions, with an average value of 31.30 mg g^−1^ dry weight. To maintain the cell turgor, the total soluble content was increased under moisture-stressed conditions. Under stressed conditions, the maximum sugar content was observed in genotype JG11 (60 mg g^−1^ dry weight) and the minimum in ICCV 201212 (34 mg g^−1^ dry weight), with a mean of 46.32 mg g^−1^ dry weight. Genotype SAGL152252 showed the maximum increase in TS content of 24-fold under moisture-stressed conditions compared to control conditions. Genotype ICCV201218 showed the minimum TS content under moisture-stressed conditions compared to control conditions (Table 3 and Figure 2C).

##### Lipid Peroxidation/Malondialdehyde (MDA) Content

MDA was significantly enhanced in all 78 chickpea genotypes under stressed conditions compared to normal conditions, and a significant difference was detected among the genotypes. Drought stress significantly increased leaf MDA content in all genotypes, ranging between 1.1 and 1.76 nmol g^−1^ DW. The maximum value was observed in genotypes SAGL162390 and SAGL 152252 (1.76 nmol g^−1^ DW), and the minimum in SAGL 22-105 (0.02 nmol g^−1^ DW), whereas under normal conditions, the maximum value was observed in genotype SAGL162390 (1.70 nmol g^−1^) and the minimum in SAGL 22-105 (1.02 nmol g^−1^ DW). The MDA content, an indicator of membrane damage due to lipid peroxidation, markedly increased in the leaves, especially in the drought-sensitive genotypes. Here, the increase ranged from 0.07-fold (SAGL152403) in the drought-sensitive genotype to 0.02-fold in the tolerant genotype (Table 3 and Figure 2D).

##### Proline Content

Proline was significantly enhanced in all 78 chickpea genotypes under stressed conditions compared to normal conditions, and a significant difference was found among the genotypes. Under normal conditions, it ranged from 13.25 to 39.85 mg g^−1^ FW, with an average of 28.59 mg g^−1^ FW, whereas under stressed conditions, it ranged from 37.12 to 70.12 mg g^−1^ FW, with a mean of 54.55 mg g^−1^ FW. Under normal conditions, the maximum amount of proline was evidenced in genotype SAGL152252 (39.85 mg g^−1^ FW), while the minimum value was recorded in genotype ICCV201102 (13.25 mg g^−1^ FW). Similarly, under stressed conditions, more proline was observed in genotype SAGL152252 (70.12 mg g^−1^ FW), but less in genotype GCP101 (37.12 mg g^−1^ FW). The proline content of the leaf increased by 13.75–32.67-fold in drought-stressed plants, with an average of 25.89-fold, including a significantly greater increase in genotypes tolerant to drought, such as SAGL162375, SAGL152314, ICCV201108, SAGL152210, and SAGL152208, and a lesser increase in sensitive genotypes, such as SAGL 22-106, ICCV 201105, and SAGL 163603 (Table 3 and Figure 2E).

##### Phenol Content

Phenol was significantly enhanced in all 78 chickpea genotypes under stressed conditions compared to normal conditions, and a significant difference was found among the genotypes. Under normal conditions, it varied between 0.57 and 1.86 mg gallic acid equivalent per g, with an average of 1.23 mg gallic acid equivalent per g, whereas under stressed conditions, it ranged from 0.74 to 2.05 mg gallic acid equivalent per g, with a mean of 1.43 mg gallic acid equivalent per g. Under normal conditions, the maximum amount of phenol was evidenced in genotype SAGL152278 (1.86 mg gallic acid equivalent per g), and the minimum in SAGL163603 (0.57 mg gallic acid equivalent per g). Similarly, under stressed conditions, a higher phenol content was observed in genotype SAGL152238 (2.05 mg gallic acid equivalent per g), and lower in SAGL163603 (0.74 mg gallic acid equivalent per g). Phenol content of the leaf increased by 0.03–1.13-fold in drought-stressed genotypes, with a mean of 0.20-fold. A significant enhancement was observed in genotype ICCV201107, which may be considered tolerant to drought, and less of an enhancement in genotype ICCV 201111, which may be considered sensitive to drought (Table 3 and Figure 2F).

### 2.3. Enzymatic Antioxidants

The generation of reactive oxygen species (ROS) in response to stress can be regulated by many antioxidant enzymes, including DPPH, H_2_O_2_, CAT, APX, SOD, and POX. To assess the survival of plants under stressed conditions, the enzymatic activities were determined.

#### 2.3.1. DPPH Content

**Figure 3:**
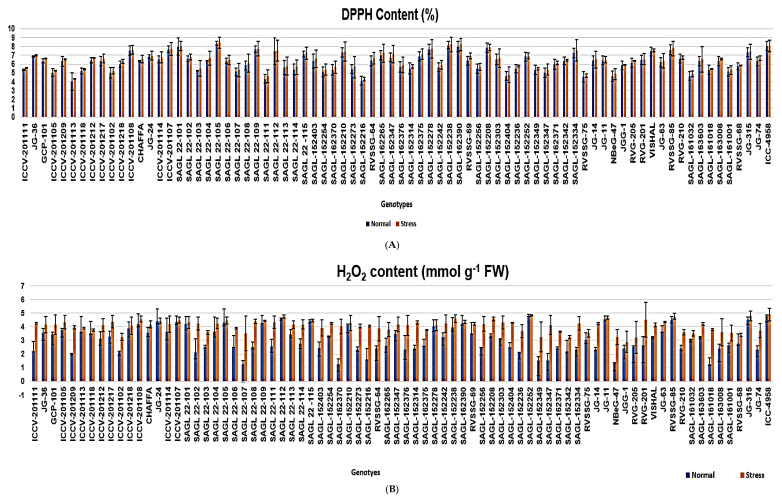

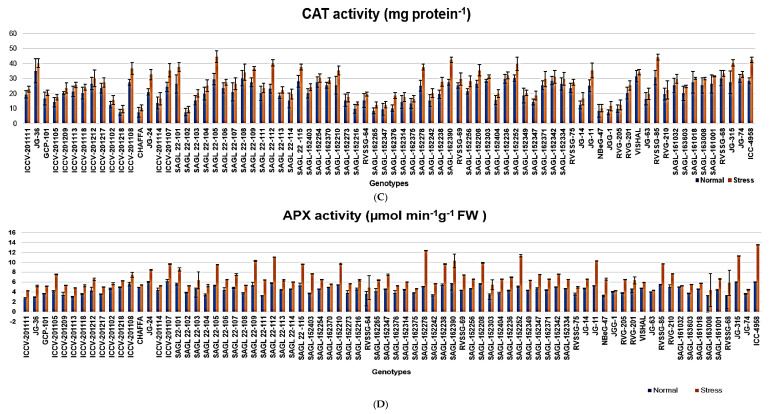

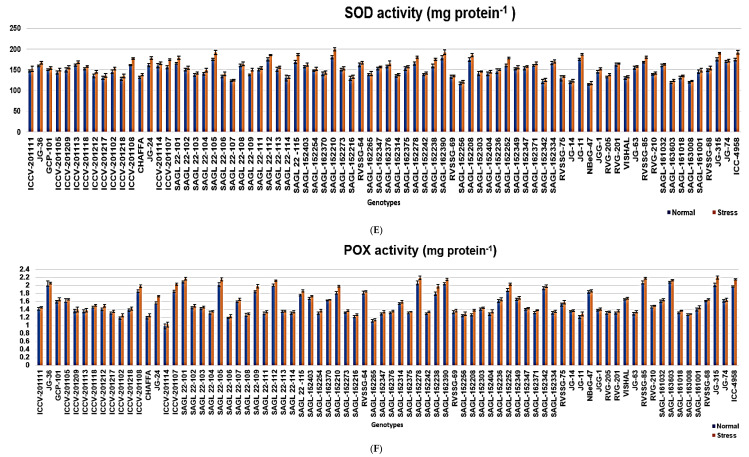
Consequence of drought stress at flowering stage on (**A**) DPPH, (**B**) H_2_O_2_, (**C**) CAT, (**D**) APX, (**E**) SOD, and (**F**) POX enzyme action of studied genotypes, where SOD, POX, CAT, and APX indicate superoxide dismutase, peroxidase, catalase, and ascorbate peroxidase, correspondingly.

DPPH content was significantly enhanced in all 78 chickpea genotypes under stressed conditions compared to normal conditions, and a significant difference was found among the genotypes. Under stressed conditions, the maximum value was found in genotype SAGL 22-105 (8.41%), and the minimum in SAGL152216 (4.26%), with an average of 6.41, whereas under normal conditions, the maximum value was found in genotype SAGL22-105 (8.32%) and the minimum in genotype ICCV201113 (4.02%), with a mean of 6.22. DPPH content in the leaf increased by 0.04–0.55-fold in drought-stressed genotypes, with an average of 0.19-fold, a significantly greater increase in genotype RVSSG69, and a lesser increase in genotypes such as ICCV201107 (0.04), ICC 4958 (0.04), JGG1 (0.05), JG315 (0.05), and SAGL152208 (Table 4 and Figure 3A).

#### 2.3.2. H_2_O_2_ Content

H_2_O_2_ content was significantly enhanced in all 78 chickpea genotypes under stressed conditions compared to normal conditions, and a significant difference was found among the genotypes. Under normal conditions, it ranged from 1.26 to 4.79 mmol g^−1^ FW, with an average of 3.05 mmol g^−1^ FW, whereas under stressed conditions, it ranged from 2.89 to 4.89 mmol g^−1^ FW, with a mean of 4.08 mmol g^−1^ FW. Under normal conditions, a higher H_2_O_2_ content was observed in genotype SAGL152252 (4.79 mmol g^−1^ FW), and lower in genotype SAGL162370 (1.26 mmol g^−1^ FW). Likewise, under stressed conditions, a higher H_2_O_2_ content was maintained in genotype ICC4958 (4.89 mmol g^−1^ FW), followed by SAGL152252 (4.82 mmol g^−1^ FW), and lower in JGG-1 (2.89 mmol g^−1^ FW). H_2_O_2_ content exhibited greater increases in drought-sensitive genotypes than in drought-tolerant genotypes. The minimum enhancement of H_2_O_2_ was observed in genotype SAGL152252 (0.03-fold), followed by JG-24 (0.04-fold), while the maximum enhancement of H_2_O_2_ was noticed in genotype SAGL162370 (2.76-fold) under stressed conditions over normal conditions. Under stressed conditions, the average enhancement of H_2_O_2_ was 0.08-fold compared to normal conditions (Table 4 and Figure 3B).

#### 2.3.3. CAT Activity

CAT has a role in the decomposition of peroxidase under drought conditions. Under drought conditions, the maximum CAT activity was revealed by genotype SAGL22-105 (44.46 mg protein^−1^) and the lowest by SAGL22-102 (9.23 mg protein^−1^), with an average of 26.48 mg protein^−1^. Nevertheless, under normal conditions, genotype SAGLJG36 (35.02 mg protein^−1^) had the highest CAT enzyme activity and genotype ICCV201218 (7.42 mg protein^−1^) had the lowest CAT enzyme activity, with a mean of 20.96 mg protein^−1^. The highest activity of the catalase enzyme was found in tolerant genotypes. The maximum enhancement of catalase was noticed in genotype SAGL22-112 (17.07-fold) under stressed conditions over normal conditions, while the minimum enhancement of catalase was evident in genotype SAGL 22-102 (1.72-fold), with a mean of 5.52 (Table 4 and Figure 3C).

#### 2.3.4. APX Activity

APX was significantly boosted in all genotypes under stressed conditions compared to normal conditions, and a significant difference was found among the genotypes. Under normal conditions, it ranged from 2.65 to 6.12 µmol min^−1^ g^−1^ FW, with an average of 4.40 µmol min^−1^ g^−1^ FW, while under stressed circumstances, it varied from 4.25 to 12.34 µmol min^−1^ g^−1^ FW with a mean of 6.90 µmol min^−1^ g^−1^ FW. Under normal conditions, the maximum APX was observed in genotype ICCV201107 (6.12 µmol min^−1^ g^−1^ FW), and the minimum in ICCV201111 (2.65 µmol min^−1^ g^−1^ FW). Similarly, under stressed conditions, a higher APX activity was recorded in genotype ICC4958 (13.52 µmol min^−1^ g^−1^ FW), followed by SAGL152278 (12.34 µmol min^−1^ g^−1^ FW), and lower in ICCV201111 and JGG-1 (4.25 µmol min^−1^ g^−1^ FW). The APX content of the leaf increased by 0.26–7.51-fold in drought-stressed plants, with an average of 2.50-fold, a significantly greater increase in genotypes tolerant to drought, such as ICCV 4958 (7.51-fold) and SAGL152278 (7.32-fold), and a lesser increase in sensitive genotypes such as SAGL161032 and JGG-1 (0.26- and 0.27-fold, respectively) (Table 4 and Figure 3D).

#### 2.3.5. SOD Activity

SOD activity was significantly enhanced in genotypes under stressed conditions compared to normal conditions, and a significant difference was evident among the genotypes. Under normal conditions, it ranged from 115 to 180 mg protein^−1^, with a mean of 148.54 mg protein^−1^, whereas under stressed conditions, it ranged from 118 to 199 mg protein^−1^, with a mean of 155.62 mg protein^−1^. Under normal conditions, a higher SOD activity was detected in genotype SAGL152210 (180 mg protein^−1^), and lower in NBeG-47 (115 mg protein^−1^). Similarly, under stressed circumstances, a higher SOD activity was maintained in genotype SAGL152210 (199 mg protein^−1^), and lower in NBeG-47 (118 mg protein^−1^). The minimum enhancement of SOD was evident in genotypes viz., JG-74, RVSSG-69, and SAGL22-107 (2-fold), and the maximum in genotype SAGL152210 (19-fold) under stressed conditions over normal conditions. Under stressed conditions, the average increase in SOD was 7.08-fold compared to normal conditions (Table 4 and Figure 3E).

#### 2.3.6. POX Activity

POX activity was significantly enhanced in all genotypes under stressed conditions compared to normal conditions, and a significant difference was found among the genotypes. Under normal conditions, it ranged from 0.98 to 2.09 mg protein^−1^, with an average of 1.52 mg protein^−1^, whereas under stressed conditions, it ranged from 1.02 to 2.20 mg protein^−1^, with an average of 1.59 mg protein^−1^. Under normal conditions, a higher POX activity was detected in genotype SAGL22-101 (2.09 mg protein^−1^), and a lower activity in ICCV201114 (0.98 mg protein^−1^). Likewise, under stressed conditions, a higher POX activity was maintained in genotype JG315 (2.20 mg protein^−1^), and lower in ICCV201114 (1.02 µmg protein^−1^). The maximum enhancement of POX was witnessed in genotype JG315 (0.19-fold), and the minimum in SAGL22-113 (0.01-fold) under stressed conditions over normal conditions. Under stress conditions, the average enhancement of POX was 0.07-fold compared to normal conditions (Table 4 and Figure 3F).

### 2.4. Correlation Analysis between Drought and Normal Conditions

**Figure 4:**
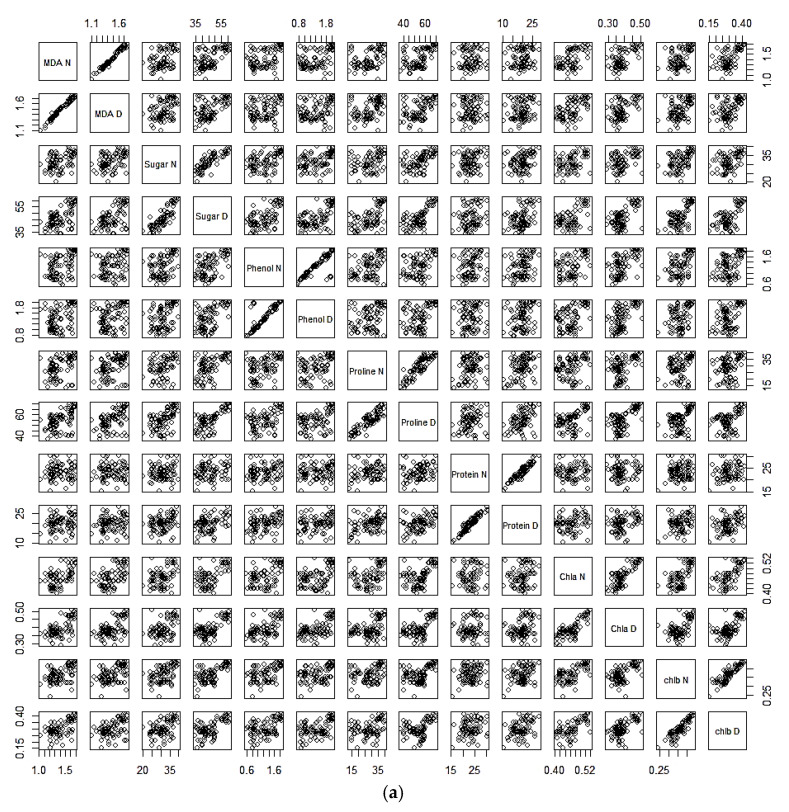

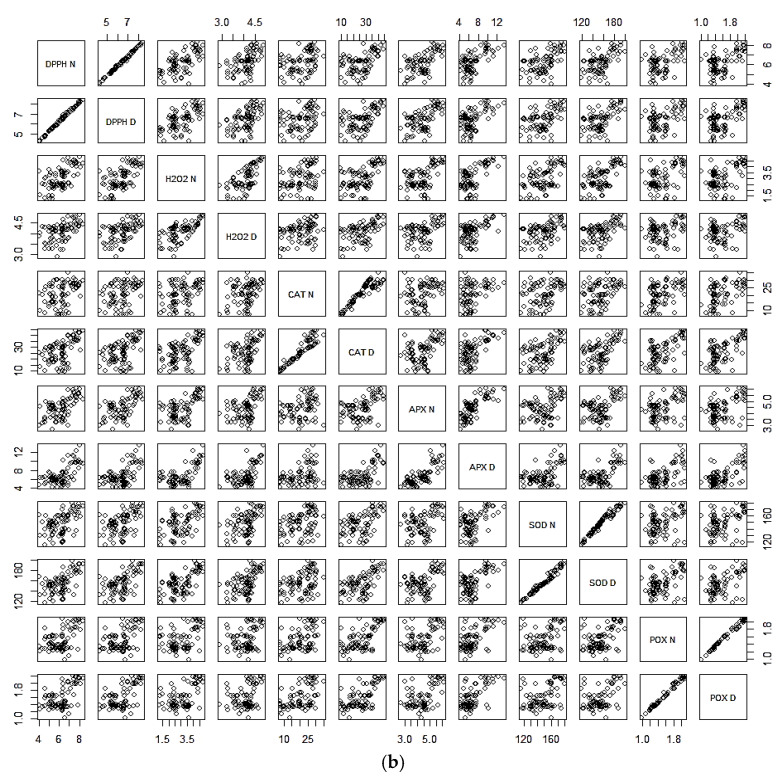
(**a**) Scatterplot matrix for non-enzymatic biochemical parameters. (**b**) Scatterplot matrix for antioxidant enzyme activity.

Under drought as well as normal conditions, there was also a positive and significant correlation between MDA, phenol, proline, chlorophyll a, and chlorophyll b. During stress, phenol, proline, chlorophyll a, and chlorophyll b revealed a positive and significant correlation with MDA. Phenol had a positive and significant correlation with proline, chlorophyll a, and chlorophyll b. Proline had a positive and significant correlation with chlorophyll a and chlorophyll b. Furthermore, there was a significant positive correlation between chlorophyll a and chlorophyll b (Table 5, Figure 4a), whereas under stressed circumstances, the correlation analysis revealed a highly positive and significant relationship between different antioxidant enzyme activities. During drought and normal conditions, there was also a positive and significant correlation between DPPH, H_2_O_2_, CAT, ascorbate, SOD, and POX. Under stressed conditions, H_2_O_2_, CAT, ascorbate, SOD, and POX revealed a positive and significant correlation with DPPH. H_2_O_2_ had a positive and significant correlation with CAT, ascorbate, SOD, and POX. CAT had a positive and significant correlation with ascorbate, SOD, and POX. Ascorbate had a positive and significant correlation with SOD and POX. Moreover, there was a significant positive correlation between SOD and POX (Table 6, Figure 4b).

### 2.5. Principal Component Analysis (PCA)

Principal component analysis was carried out for all genotypes, as well as the seven non-enzymatic and six enzymatic biochemical parameters under investigation in each treatment, to better understand the relationship between genotypes and to extract the significant and valuable information present in the data matrix. Additionally, the principal component analysis reduced the number of qualities that accounted for the highest proportion of variability found in the data matrix. More than one eigen value in the major components accounted for about 10% of the overall variation. The principal components indicated that the higher eigenvalues were the most representational of system features [22].

**Figure 5:**
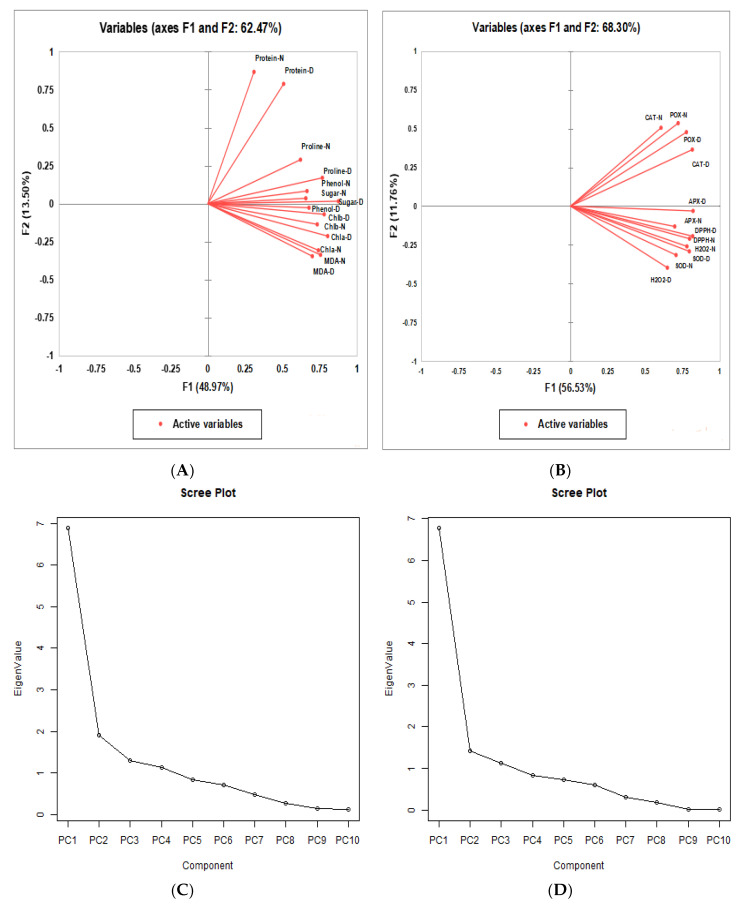
(**A**,**B**) Biplot of chickpea genotypes for the first four principal components. Based on the angle between the traits, the biplot was categorized into four groups. (**C**,**D**) Scree plot representing cumulative variability and eigenvalues for studied non-enzymatic and antioxidant activities for biochemical parameters.

PC-I, PC-II, PC-III, and PC-IV had eigen values > 1 out of the 14 principal components, according to the scree plot of the non-enzymatic biochemical parameters (Figure 5C) Eigen values < 1 for the remaining principal components indicated that they were not further discussed. The first four components were the most significant; separately, PC-I, PC-II, PC-III, and PC-IV contributed 49.16%, 13.57%, 9.27%, and 8.01% of the overall variability in this investigation under various biochemical parameters, respectively. Combined, both components provided 80.01% of the variability (Table 7).

For a more reliable identification of the genotype with the maximum value for one or more traits, genotype-by-trait (GT) biplots were constructed for PC-I, PC-II, PC-III, and PC-IV for all genotypes and all traits under all treatments. These exhibited the trait profile of a genotype. In the biplots, to understand the interrelationship among the genotypes and traits, vector lines were drawn from the origin of the biplots. Genotypic performance (how it differs from the average genotype) can be estimated by the distance of a genotype from the origin of the biplot; distant genotypes could have maximum values for one or more traits. The Pearson correlation between traits was indicated by the cosine angle between the two traits, i.e., no correlation = angle of 90°; positive correlation =< 90°. Based on the angle between the vectors, the biplot was categorized into two major groups: Group 1 showed a positive correlation between protein and proline; Group 2 depicted a positive correlation among MDA, chlorophyll a, and chlorophyll b (Figure 5A). The scree plot of biochemical antioxidant enzyme activities (Figure 5C,D) showed that, among the 12 principal components, PC-I, PC-II, PC-III, and PC-IV had extracted eigenvalues > 1. The remaining principal components had eigenvalues < 1, so they were not investigated further. The first four components were evidently the most influential: PC-I, PC-II, and PC-III individually contributed 56.41%, 11.83%, and 9.33% of the total variability, respectively, while cumulatively, they contributed 77.57% of the variability in this study under different biochemical parameters. Based on the angle between the vectors, the biplot was categorized into two major groups: Group 1 showed a positive correlation between CAT and POX; Group 2 depicted a positive correlation among DPPH, H_2_O_2_, and SOD (Figure 5B).

## 3. Discussion

The drought stress that developed during seed filling had a substantial effect on chickpea growth and productivity. According to Reddy et al. [23] and Yadav et al. [24], plants that thrive in low-water conditions exhibit physiological and biochemical changes that may constitute pathways for water deficit adaptation. Growth inhibition is often associated with altered plant water status and a decrease in the relative water content of leaves [25]. Drought tolerance critically depends on plants’ physiological ability to maintain the operation of their photosynthetic system in the face of water stress [26]. Additionally, changes in plant metabolism brought on by environmental stressors such water scarcity cause ROS to build up in the cells, which oxidatively damages the plants [27,28]. A shortage of water in the chickpea crop alters several physiological and biochemical processes [2].

The current study shows that several physiological indicators drastically decreased because of drought stress. The average performance of several characteristics under both normal and drought-prone conditions showed that the mean of most of the characteristics under study had sufficiently decreased. A plant’s water potential is determined by calculating its relative water content. Our investigation revealed a noteworthy decrease in RWC across all genotypes analyzed. Under typical circumstances, genotype SAGL 22-102 displayed a high RWC. However, genotype SAGL152278 showed a greater RWC under stressful conditions. The relative water content drastically dropped when drought stress was applied. Under moisture stress, SAGL161001 showed the largest loss in RWC among the sensitive genotypes, while SAGL15221 showed the least reduction among the tolerant genotypes. Because tolerant genotypes have more effective control mechanisms to maintain cell and tissue hydration under water stress by regulating stomatal opening, their RWC declines less than that of sensitive genotypes. When compared to the control plants, the plants under drought stress had a considerably lower RWC. Rizvi et al. [29] and Sharma et al. [16] have similarly reported these changes in RWC in response to water stress. All chickpea genotypes included in our investigation had a significant increase in SWD. Genotype SAG162375 exhibited a substantial SWD in both scenarios. Under stressed conditions compared to normal conditions, genotype SAGL152347 (0.97) showed the lowest rise in SWD, while JG-14 (16.53) showed the highest increase in SWD. SWD values were significantly greater in the susceptible genotypes, but lower in the tolerant genotypes when compared to the susceptible genotypes. A rising water deficit in a variety of crops has also been linked to a similar trend of a considerable increase in SWD. When comparing drought-stressed conditions to normal conditions, CTD reduced [15,30]. A higher CTD was shown in genotype SAGL162370 under normal circumstances and in genotype SAGL152303 under stressful circumstances. Under stressed conditions compared to normal conditions, genotype SAGL22-115 showed the lowest drop in CTD (0.42), while genotype SAGL162371 showed the largest reduction in CTD (7.50). According to Karimizadeh et al. [31] and Shakeel et al. [32], the drought-tolerant chickpea genotypes showed a higher CTD under drought-stressed circumstances than other genotypes, demonstrating their remarkable capacity to maintain a canopy cooler than the others. In early-generation selections, CTD has already been used as a selection indication for tolerance to drought and high-temperature stress. Karimizadeh et al. [33] and Tiwari et al. [2] also reported results that were similar. Higher MSI values in germplasm lines indicate a greater capacity to withstand drought stress [34,35]. When comparing drought-stressed settings to normal conditions, MSI was lower. Genotype JG11 was shown to have a higher MSI under normal circumstances, which was maintained during stress. Under challenged conditions compared to normal conditions, genotype SAGL162389 (14.10) showed the greatest reduction in MSI.

When a crop faces stressed conditions, the activities of antioxidant enzymes are altered for cellular protection. Plant cells defend against oxidative stress by lowering ROS concentrations while retaining antioxidant defense chemicals and osmolytes. Drought stress, alone or in amalgamation, causes an overproduction of reactive oxygen species in many organelles, creating a danger to cellular metabolic functions [15,36,37]. These harmful reactions damage biological components, for instance, the photosynthetic system and thylakoidal membranes, resulting in DNA larceny and amino acid and protein oxidation [38,39,40,41,42]. Drought tolerance mechanisms in the chickpea relate to the increased synthesis of osmolytes and antioxidants, which aid in metabolism, preserve macromolecules, and maintain membrane veracity, resulting in drought adaption.

The current study examined the effects of proline content, lipid peroxidation, total soluble sugar, and protein content on osmotic adjustment. For plants, the primary functions of the chlorophyll pigments are light absorption and tumbling abilities. Because of the unwavering relationship between chlorophyll concentration and photosynthetic capability, a genotype’s comparative tolerance can be regulated by the relative amounts of each gene. The results showed that genotypes SAGL162371 and SAGL201108 under stressful and normal conditions, respectively, produced the highest amounts of Chl_a_ and Chl_b_. Drought stress meaningfully reduced leaf chlorophyll a and chlorophyll b in all genotypes, especially in the drought-sensitive genotypes such as SAGL162370, SAGL22-107, and SAGL163603, and exerted minimum changes in tolerant genotypes such as ICCV201218, ICCV201108, SAGL22-115, SAGL152403, SAGL162265, and ICCV4958. The reduction in leaf chlorophyll a and chlorophyll b in drought-sensitive genotypes was also reported by Rizvi et al. [29], Kumar et al. [43], Jameel et al. [41], and Sahu et al. [44]. Drought-induced decay in photosynthesis could also result in a decrease in the carbon skeleton of amino acids, leading to a reduction in protein synthesis [45]. The decrease in protein content under stressed conditions also agree with earlier studies, including those by Rizvi et al. [29], Bhagyawant et al. [46], Shah et al. [35], Jameel et al. [41], and Sahu et al. [44]. Drought tolerance is usually related to the gathering of osmo-protectants such as proline, for example [47]. Proline content was assessed under both normal and stressed conditions. Under both circumstances, the maximum proline content was observed in genotype SAGL152252. There was a substantial upsurge in proline content in both tolerant and sensitive genotypes under stressed conditions. Drought-tolerant genotypes had a significant increase in proline content, including SAGL162375, SAGL152314, ICCV201108, SAGL152210, and SAGL152208, while less of an increase was evidenced in sensitive genotypes such as SAGL22-106, ICCV201105, and SAGL163603. This suggests that the tolerant genotype has a basic mechanism to fight alterations in water status in its environment by regulating its proline concentration. Under drought-stressed conditions, the manufacturing of proline aids plants in the alteration of their osmotic cell potential to preserve cell turgor, which contributes to drought tolerance. The direct indication of the function of proline under stress has been addressed by, Bhagyawant et al. [46], Jameel et al. [41], Sahu et al. [44], and Rajput et al. [48]. Among the non-enzymatic antioxidants, total phenolic content (TPC) represents the foremost bio-active compounds, which accomplish innumerable structural tasks in the body and are directly related to antioxidant activity [49]. The tolerant genotype showed a higher upsurge in phenol content than the sensitive genotypes. In the case of normal conditions, the maximum phenol content was produced in genotype SAGL152278. Similarly, under stressed conditions, a higher phenol content was recorded in genotype SAGL152238. Significant enhancement was observed in genotype ICCV201107, which may be considered tolerant to drought, and less of an enhancement in genotype ICCV201111, which may be considered sensitive to drought. A higher phenol content under stressed conditions was also reported by Sahu et al. [44]. Changes in the magnitude of soluble sugars in relation to drought stress may be due to increased sugar production, the alteration of carbohydrates from storage forms to soluble sugars, the breakdown of cell wall polysaccharides, and changes in the rate of sugar transport. This results in reduced water potential due to the breakdown of starch by hydrolytic enzymes, known as amylases, into glucose and maltose, which elevates the osmotic concentration of the cell [50]. Consequently, cellular turgor, expansion growth, and the uptake of water and minerals through the roots are sustained. In the present investigation, increased TSS levels were evidenced in genotype SAGL22-101 under normal conditions, and JG11 had a high sugar content under stressed conditions. Genotype SAGL152252 showed the maximum increase in TSS content under moisture-stressed conditions compared to control conditions. Similarly, an increased level of total soluble sugar was reported under stressed conditions in earlier studies by Jameel et al. [41] and Rajput et al. [48]. MDA content is employed as a lipid peroxidation indicator in investigations related to oxidative stress and usually indicates injury in plant membranes [51]. In this research, we found that in general, the MDA content was enhanced under stress in all the genotypes. The maximum value was recorded in genotypes SAGL162390 and SAGL152252 under stressed conditions, whereas under normal conditions, the maximum value was observed in genotype SAGL162390. Here, the maximum increase in MDA was observed in genotype SAGL152403, which may be considered a drought-sensitive genotype, while the minimum increase was in genotype SAGL 22-105, which may be considered a tolerant genotype. MDA can play a positive role in acclimation processes by energizing the governing genes involved in plant defense apparatus. Analogous results of increased MDA content were reported under water-stressed conditions in the chickpea by Jameel et al. [41] and Rajput et al. [48].

Increased activity of SOD, POX, CAT, and APX was observed in all genotypes under drought stress compared to normal conditions. A higher activity level was noticed in tolerant genotypes compared to other genotypes. Drought stress triggered an increase in the DPPH content in the leaves of all genotypes. Under stressed conditions, the maximum value was found in genotype SAGL22-105, whereas under normal conditions, the maximum value was recorded in genotype SAGL22-105. A significant increase was recorded in genotype RVSSG 69, and a lesser increase in genotypes including ICCV201107, ICC4958, JGG1, JG315, and SAGL152208. A higher DPPH content under stressed conditions was also reported earlier by Saleghi et al. [52]. ROS including hydrogen peroxides, superoxidase anions, and hydroxyl radicals are byproducts of physiological metabolism. This metabolism is a part of the defense mechanism against drought stress. Drought-stress-induced stomatal closure favors photorespiration and the production of H_2_O_2_ in peroxisomes. H_2_O_2_ content was meaningfully enhanced in all genotypes under stressed conditions compared to normal conditions. Under normal conditions, a higher H_2_O_2_ content was evidenced in genotype SAGL152252. Similarly, under stressed conditions, a higher H_2_O_2_ content was maintained in genotype ICC4958. H_2_O_2_ content had greater increases in drought-sensitive genotypes than in drought-tolerant genotypes. The minimum enhancement of H_2_O_2_ was observed in genotype SAGL152252, followed by JG-24, under stressed conditions over normal conditions. Similar results of increased H_2_O_2_ content were reported under water-stressed conditions in the chickpea by Bhagyawant et al. [46] and Jameel et al. [41].

Catalase eliminates H_2_O_2_ by breaking it down into H_2_O and O_2_ [53]. CAT has a role in the decomposition of peroxidase under drought conditions and plant drought tolerance [53]. Under drought conditions, the maximum CAT activity was demonstrated by genotype SAGL22-105, while under normal conditions, genotype SAGLJG36 had the highest CAT enzyme activity. A higher activity of the catalase enzyme was found in the tolerant genotypes. The maximum enhancement of catalase was noticed in genotype SAGL22-112 under stressed conditions over normal conditions, while the minimum enhancement of catalase was observed in genotypes viz., SAGL 22-105, SAGL 22-112, SAGL22-105, SAGL152208, SAGL152278, ICC4958, and JG315. Several researchers reported a similar increased level of catalase activities under water-stressed conditions in the chickpea including Jan et al. [54] and Jameel et al. [41]. APX is the most imperative peroxidase that aids in H_2_O_2_ scavenging, acting as an electron donor and defending cell components by eradicating ROS [55]. In the current study, the increase in APX activity indicated its inactivation by the accumulated H_2_O induced by water shortage. Under normal conditions, the maximum APX was observed in genotype ICCV201107 and the minimum in ICCV201111. Genotypes such as SAGL152252, SAGL 22-105, SAGL22-112, ICCV4958, JG315, and SAGL152278, with a significantly greater increase in APX, showed tolerance to drought. These genotypes displayed drought tolerance as they portrayed enhanced enzymatic antioxidant potential. Parallel results were also reported in the chickpea under drought stress by [41,54], as they observed that increased antioxidant activity constitutes the first line of defense via the detoxification of superoxide radicals to H_2_O_2_. Increased levels of SOD activity quench higher levels of superoxide radicals generated under drought stress. Under both conditions, a higher SOD activity was observed in SAGL152210. The minimum enhancement of SOD was observed in JG-74, RVSSG-69, and SAGL 22-107, while the maximum enhancement of SOD was noticed in SAGL152210, ICCV201108, SAGL 22-105, SAGL162390, SAGL 22-112, and SAGL22-109 under stressed conditions over normal conditions. The enhancement of SOD activity in drought-tolerant cultivars of the chickpea was also reported by some other researchers [41,54]. Peroxidases (PODs) are enzymes that catalyze an oxidation–reduction reaction, employing free radicals that convert several compounds into the polymerized or oxidized form [56]. Under normal conditions, a higher POX activity was observed in SAGL 22-10. Similarly, under stressed conditions, a higher POX activity was maintained in genotype JG-315. The maximum enhancement of POX was noticed in ICC4958, JG315, SAGL152252, SAGL162370, SAGL152238, SAGL162390, SAGL152208, and ICCV201108 under stressed conditions over normal conditions. The increase in peroxidase activity under drought conditions has also been reported by Jan et al. [54] and Jameel et al. [41].

The PCA-based biplot is the most valuable multivariate analysis to study the trait interactions and genotypic performance of crops under stressed conditions [41,54]. The PCA biplot analysis performed for both enzymatic and non-enzymatic biochemical traits demonstrated a good contribution with respect to the performance of genotypes by depicting the associations between the studied traits and the allocation patterns of the studied genotypes under stressed conditions. PCA biplots of non-enzymatic biochemical traits demonstrated a strong positive correlation between MDA, protein, proline, chlorophyll a, and chlorophyll b, while the PCA biplots of the enzymatic biochemical traits, DPPH, H_2_O_2_, CAT, SOD and POX, displayed a sturdy positive association with the investigated enzymatic biochemical parameters. These strongly positively associated traits may be considered to have a maximum contribution to yield output in comparison to other studied traits. In our research, the genotypes viz., SAGL22-115, ICC4958, ICCV201108, ICCV201107, SAGL152252, and JG 11 were selected for their ability to tolerate drought based on non-enzymatic biochemical characteristics. The genotypes SAGL152208, SAGL22-105, SAGL22-112, ICC201108, SAGL152278, SAGL152252, SAGL162371, SAGL162390, ICC4958, and JG315 may be considered drought-tolerant based on antioxidant enzymatic activities. PCA biplots were successfully utilized in several recent studies for the prioritization of multivariate traits and to distinguish effective genotypes under drought-stressed conditions [57].

## 4. Materials and Methods

In the experimental field, a total of 78 genotypes of chickpeas (Table 8 were sown in a randomized block design with three replications under stressed and irrigated circumstances (control). The experimental trial was conducted in the field of the Department of Genetics and Plant Breeding, College of Agriculture, RVSKVV, Gwalior M.P., India. In November of 2022, the crop was sown. Four 3 m long rows with 30 cm row spacing were used to accommodate each genotype. Physiological parameters such as relative water content, saturated water deficit, membrane stability index, and depression in canopy temperature were measured from leaves during the reproductive stage. Biochemical parameters included protein content, total soluble sugar, lipid peroxidation, proline, superoxide dismutase, peroxidase and catalase activities, H_2_O_2_ content, ascorbate peroxidase, and DPPH parameters. The recognized agronomic practices were adopted throughout the crop season for proper crop growth and development. The crop was maintained free from weeds, diseases, and pests by applying suitable plant protection methods. Under normal conditions, irrigation was performed as per need, while no irrigation was provided after the initial stage under stressed conditions.

### 4.1. Physiological Parameters

From each treatment, three plants were randomly selected for the recording of different physiological traits.

### 4.2. Relative Water Content (RWC)

To analyze relative water content, the plants were sampled at mid-day (between 9:00 a.m. and 11:00 a.m.), and the third completely developed leaves from the top were taken. They were then promptly packed in humified polythene bags, brought to the laboratory (on ice), and promptly weighed to determine their fresh weight. After that, the leaf was stored for three hours in a Petri dish containing distilled water. The leaf, which was now completely turgid, was then weighed once more and baked for 72 h at 65 °C until it reached a steady dry weight. Using the following formula provided by Weatherley [58], RWC (%) of leaves was computed.

RWC (%) = (Fresh weight − dry weight)/(Turgid weight − dry weight) × 100

### 4.3. Saturation Water Deficit

SWD was calculated based on following formula.

SWD = 100 − RWC

### 4.4. Canopy Temperature Depression (at Reproductive Stage)

Canopy temperature indirectly measures plant transpiration and plant water status. Using an infrared thermometer, the canopy temperature of an extended second leaf was measured at 60 and 90 DAS from the tip of the main stem between 11:00 a.m. and 12:00 noon. Three tagged plants were used to obtain the mean of five readings per plot. The same position (distance, angle) was maintained throughout the measurement. Weather that was windy or cloudy was avoided. Furthermore, the measurements were taken at noon with the sun behind the operator.

### 4.5. Membrane Stability Index

Two sets of 200 mg of leaf sample were used to determine the membrane stability index (MSI) in a test tube holding 10 mL of double-distilled water. To measure the electrical conductivity (C_1_) of the solution using an electrical conductivity meter, one set was heated to 40 °C for thirty minutes in a water bath. To measure the second set’s conductivity (C_2_), it was heated to 100 °C for ten minutes in a boiling water bath, as previously mentioned. To determine the membrane stability index, the following formula given by Khanna-Chopra and Selote [59] was used.

MSI = [1 − {C_1_/C_2_}] × 100

where C_1_ is the electrical conductivity of water containing the leaf sample in set one and C_2_ is the electrical conductivity of water containing the leaf sample in set two.

### 4.6. Biochemical Traits

All biochemical analyses including enzymatic and non-enzymatic activities were performed at Biochemical Analysis Laboratory, Department of Plant Molecular Biology and Biotechnology, College of Agriculture, RVSKVV, Gwalior, M.P., India. The chemicals utilized in the biochemical analysis were procured from Cisco, Himedia, and Sigma. All reagents were of analytical grade or higher, with their purity levels conforming to the specific requirements outlined in standard biochemical analysis guidelines.

### 4.7. Chlorophyll Content

Total chlorophyll was calculated as per the method given by Arnon et al. [60]. One hundred mg of fresh leaf sample was randomly taken after 70 days from sowing. Then, the leaf sample was finely crushed in 10 mL of 80% acetone and transferred into a Falcon tube followed by centrifugation (Centurion, Scientific limited, Refrigerated) for 15 min at 10,000 rpm, and the green supernatant was transferred into a fresh 15 mL Falcon tube. Readings were taken in a spectrophotometer at 645 nm, 663 nm, and 470 nm wavelengths.

### 4.8. Estimation of Proline Content

Proline was measured spectrophotometrically using the method of Bates et al. [61], the ninhydrin technique. First, 0.25 mg of a random fresh leaf sample was taken after 70 days from sowing. The leaf sample was very finely crushed in 3.0 mL 3% homogenize sulfosalicylic acid solution in a mortar pestle followed by centrifugation at 1000 rpm for 15 min, and the supernatant was transferred to a 15 mL Falcon tube. Then, 2.0 mL of supernatant was taken from the Falcon tube and mixed with 2.0 mL ninhydrin acid (ninhydrin + glacial acetic acid). Subsequently, it was heated at 100 °C for 60 min in a water bath, and then the mixture (ice bath) was cooled until reaching room temperature, 25 °C. Finally, 4.0 mL of toluene was supplemented until a pink layer appeared, then the upper layer was taken and the reading was recorded at 520 nm absorbance in a spectrophotometer. The proline concentration was determined from a standard curve using D-proline.

### 4.9. Estimation of Sugar Content (mg g^−1^ Fresh Weight)

Sugar content was estimated by employing the anthrone reagent method as described by Dubois et al. [62]. One hundred mg of a random fresh leaf sample was taken from the field after 70 days from sowing and crushed in 5.0 mL of 80% ethanol in a mortar and pestle until the leaf completely disappeared and a fine liquid solution was made, which was poured into a 15.0 mL Falcon tube and centrifuged at 1000 rpm for 10 min. Then supernatant was transferred to a fresh 15.0 mL Falcon tube and an additional 5.0 mL of 80% ethanol was added to the old tube, which was again centrifuged at 1000 rpm for 10 min. The supernatant was transferred to the Falcon tube. A total of 10 mL (5.0 mL + 5.0 mL) of supernatant was heated in a glass bottle in an oven at 65 °C until it dried. After drying, 1.0 mL of distilled water was added. Then, 100 microliters were taken from the glass bottle with the help of a pipette, and then the anthrone reagent was added to the Falcon tube. The Falcon tube was heated at 100 °C for 30 min and cooled until it reached room temperature, and then the absorption was measured at the 630 nm wavelength in a spectrophotometer. A series of standard glucose solutions (e.g., 0.2, 0.4, 0.6, 0.8, 1.0 mg/mL) were prepared and treated in the same manner as the samples. The absorbance was plotted against the concentration to create a standard curve.

### 4.10. Lipid Peroxidation Assay

The method of Hodges et al. [63] was employed to measure the lipid peroxidation in terms of MDA content. Twenty-five mg of leaf sample was crushed in liquid nitrogen. Five hundred microliters of 0.1% trichloroacetic acid were added before vortexing and centrifugation at 10,000 rpm for 10 min. A total of 100 µL of supernatant was taken in an Eppendorf tube and 200 µL of 0.5% TBA was added. The reaction mixture was heated to 95 °C for 30 min and quickly cooled to −80 °C, holding for 2 min to stop the reaction. After 2 min, it was centrifuged at 10,000 rpm for 10 min and the supernatant was taken for reading at the 532 nm wavelength. The MDA content was calculated by using an extinction coefficient of 155 mM^−1^ cm^−1^.

The MDA concentration was calculated according to the following formula.

6.45 × (A532 − A600)/155

### 4.11. Extraction and Estimation of Total Protein

The protein content was calculated using the Lowry et al. [64] technique. A pestle and mortar were used to macerate 500 mg of plant materials with 10 mL of 20% trichloroacetic acid. The homogenate was centrifuged at 600 rpm for 15 min. The supernatant was discarded. After adding 5.0 mL of 0.1 N NaOH to the pellet, it was centrifuged for 5 min. The supernatant was preserved and added to 0.1 N NaOH to make 10 mL. The amount of protein was estimated using this extract. Initially, 5 mL of reagent ‘C’ were added to one mL of the extract in a 10 mL test tube. After mixing the solution, it was left in the dark for 10 min. Following the addition of 0.5 mL of Folin phenol reagent, the mixture was exposed to darkness for 30 min. The UV spectrophotometer read the sample at 660 nm. The protein content was given on a fresh weight basis in mg g^−1^. The standard curve was prepared using bovine serum albumin (BSA) as the standard protein.

### 4.12. Estimation of Phenol Content

The total phenol content was determined using the Swain and Hillis [65] technique. Under alkaline conditions, phenol lowers phospho-tungstate molybdic acid to form a blue color complex that may be detected using calorimetry. A single gram of oven-dried and powdered seed was obtained, and 20 mL of 80% alcohol was added. The mixture was then centrifuged at 10,000 rpm for 15 min, and the supernatant was gathered in three test tubes for the biological, standard, and blank samples. In a test tube, 1.0 mL of phenol reagent and 1 mL of supernatant were combined. Next, 2.0 mL of sodium carbonate solution was added, and the volume was adjusted with 50 mL of distilled water. Using the same 1.0 mL gallic acid solution, blank and standard solutions were also made using the aforesaid procedure. All three test tubes were filled, and the mixture was then allowed to continue to incubate at room temperature. Following the procedure, the color intensity was measured at the 650 nm wavelength, and the total phenol content was computed in terms of mg g^−1^ of sample using a standard curve made of gallic acid.

### 4.13. Enzymatic Antioxidants

Under drought stress, plants have evolved a robust antioxidant mechanism to counteract oxidative damage. It involves the actions of antioxidant enzymes, which tightly control the production of ROS and their uptake in various plant compartments.

### 4.14. Procedure for the Extraction of Enzymes

After 10 DAS, leaf samples (250 mg) were collected and processed to a fine powder using liquid nitrogen. Before centrifuging at 10,000 rpm for 15 min at 4 °C, the ground powder was homogenized in 1.5 mL of ice-cold extraction solution that contained phosphate buffer (100 mM, pH 7.0), 1% PVP, and 1 mM EDTA. After being separated, the supernatant was kept at 4 °C until the spectrophotometer was used to evaluate the enzymatic activity.

### 4.15. Estimation of DPPH (1, 1-Diphenyl-2-picryl hydrazyl) Radical Scavenging-Method

The DPPH assay was performed following the procedure developed by Sanja et al. [66]. Using 10 mL of acidified methanol, 10 mg of chickpea seed flour was added. After 20 min in a water bath, the sample solution was heated to 40 °C. The final blend was centrifuged for 20 min at 2500–3000 rpm. The result was three separate extracts, one for each genotype. In a test tube, 100 μL of sample extract was extracted and diluted to 2.9 mL with pure methanol to assess the reduction of the DPPH radical. After that, this sample mixture was combined with 150 μL of DPPH solution, which acted as a control with a comparable concentration (4.3 mg in 3.3 mL of methanol). For 15 min, the resultant sample solution was left to stand at room temperature in the dark. After 15 min, the mixture was agitated and the absorbance at the 515 nm wavelength was measured using a UV/VIS spectrophotometer. Using the following formula, the percent (%) free radical-scavenging activity was determined:

Percent (%) free radical-scavenging activity = Control absorbance − Sample absorbance × 100/Control absorbance

where, control absorbance is the absorbance of the DPPH solution without the extract.

### 4.16. Estimation of Hydrogen Peroxide (H_2_O_2_, mmol g^−1^ FW)

Hydrogen peroxide was estimated as per the method described by Alexieva et al. [67]. Initially, 25 mg of leaf sample was taken and crushed in liquid nitrogen. Five hundred µL of 0.1% trichloroacetic acid was added and vortexed followed by centrifugation at 10,000 rpm for 10 min. Then, 100 µL of supernatant was taken in a microcentrifuge tube and 200 µL of 0.5% TBA was added. The reaction mixture was heated at 95 °C for 30 min and quickly cooled to −80 °C, holding for 2 min to stop the reaction. After 2 min, it was centrifuged at 10,000 rpm for 10 min and the supernatant was taken for reading at 532 nm absorbance. A series of hydrogen peroxide standards were prepared to create a calibration curve. H_2_O_2_ content, expressed as nmol g^−1^ fresh weight, was determined based on the standard curve generated from known concentrations of H_2_O_2_.

### 4.17. Estimation of Catalase Activity

The Catalase Activity Protocol [68] was followed to measure the enzyme’s activity. Using a cold pestle and mortar, 100 mg of plant leaf samples from stressed and normal plants were obtained and homogenized in 5 mL of 0.1 M phosphate buffer (pH 6.4). The crude extract was centrifuged for 20 min at 4 °C and 10,000 rpm. Until the enzymatic test was finished, the enzyme extract was kept in a low-temperature storage area. Using 2.6 mL of 0.1 M phosphate buffer (pH 6.4), 0.1 mL of enzyme extract, and 0.1 mL of 1.0% H_2_O_2_, the enzyme’s activity was measured. At room temperature, the reaction mixture was rapidly combined. Similar preparations were made for a blank, except that the reaction mixture received 0.1 M phosphate buffer (pH 6.4) in place of the enzyme extract. For two minutes, changes in absorbance at 230 nm were recorded at 15 s intervals.

### 4.18. Estimation of APX Activity

The method developed by Nakano and Asada [69] was used to measure APX activity. Diluted enzyme extract (20 µL) was added to 50 mM potassium phosphate buffer (880 µL) containing 0.5 mM ascorbate to form the reaction mixture to measure APX activity. The addition of 1 mM H_2_O_2_ (100 µL) initiated the process. For two minutes, decreasing absorbance was measured at 290 nm at 15 s intervals.

### 4.19. Estimation of SOD Activity

Twenty-five milligrams of crushed leaf material were placed in a microcentrifuge tube, filled with 250 µL of 0.1% trichloroacetic acid, centrifuged for 20 min at 10,000 rpm, and allowed to settle. Next, 160 µL of phosphate buffer was added to 160 µL of supernatant in an Eppendorf tube. Next, 1 M potassium iodide in 680 µL was added. After an hour in the dark, the reaction mixture was analyzed for absorbance at 390 nm.

### 4.20. Estimation of POX Activity

The assay for peroxidase activity was carried out in accordance with Castillo’s recommended methodology [70]. Using a cold pestle and mortar, 100 mg of leaf samples from stressed and normal plants were homogenized in 5.0 mL of 0.1 M phosphate buffer (pH 6.4) to prepare the sample for enzyme extraction. The crude extract was centrifuged for 20 min at 4 °C and 10,000 rpm. Until the enzyme assay was carried out, the supernatant was kept at 4 °C. Then, 4.6 mL of 0.1 M phosphate buffer (pH 6.4), 0.2 mL of pyrogallol (50 µM), 0.1 mL of 50 µM H_2_O_2_, and 0.1 mL of enzyme extract were added to create the reaction mixture. The mixture was incubated for five minutes at 25 °C. After that, 0.5 mL of 5.0% H_2_SO_4_ was added to stop the reaction. Absorbance was measured at 420 nm with the help of spectrophotometer.

### 4.21. Statistical Analysis

Using SPSS V20 software (SPSS, IBM crop., Armonk, NY, USA), data were subjected to analysis of variance (ANOVA). Line diagrams based on means were created to examine the genotype’s response to various treatments. Using STAR V2.0.1 (IRRI, Los Baños, Philippines) and SPSSV20 software, respectively, the significance was determined by means of analysis of variance (ANOVA) and Duncan’s multiple range test (DMRT) at *p* < 0.05. Using the STAR V2.0.1 program, biplots were created for the first four principal components of the principal component analysis (PCA) and genotypic selection under various situations. The same program was used to perform cluster analysis and correlation (Pearson test) for all genotypes under both conditions using algometric hierarchical clustering.

## 5. Conclusions

In the present investigation, we found that chickpea plants exhibit a range of drought stress adaptation mechanisms, which include simple morphological to physiological or biochemical characteristics that function as significant stress tolerance markers. Genotypes viz., SAGL152208, SAGL22-105, SAGL22-112, ICC201108, SAGL152278, SAGL152252, SAGL162371, SAGL162390, ICC4958, and JG315 may be considered drought-tolerant lines. The selected potential cultivars may be tested in other agroclimatic zones before being released directly or used in national and international hybridization programs to enlarge the genetic basis of cultivated gene pools. The promising line(s) with drought-tolerant characteristics can easily fit in India’s rainfed chickpea regions.

## Figures and Tables

**Table 1:** Mean physiological parameters for chickpea genotypes under normal and stressed conditions.

Genotype	RWC (%)			CTD (°C)			SWD (%)			MSI (%)		
	Normal	Drought	Reduction	Normal	Drought	Reduction	Normal	Drought	Increase	Normal	Drought	Reduction
ICCV-201111	43.19 ^b–i^	39.43 ^a–i^	3.76	15.2 ^a–i^	12.32 ^a–g^	2.88	56.81 ^y–Vi^	60.57 ^t–I^	3.76	54.36 ^a–l^	47.28 ^a–m^	7.08
JG-36	43.96 ^b–k^	39.84 ^a–j^	4.12	16.92 ^a–l^	14.52 ^a–g^	2.4	56.04 ^v–VI^	60.16 ^s–I^	4.12	65.68 ^r–I^	57.23 ^a–o^	8.45
GCP-101	56.61 ^n–III^	50.11 ^i–v^	6.50	19.64 ^a–l^	15.48 ^a–g^	4.16	43.39 ^d–s^	49.89 ^d–v^	6.50	57.46 ^b–r^	50.33 ^a–o^	7.13
ICCV-201105	63.44 ^x–V^	61.36 ^w–y^	2.09	27.42 ^b–l^	20.12 ^a–g^	7.3	36.56 ^b–h^	38.64 ^a–f^	2.09	54.40 ^a–l^	43.29 ^a–i^	11.11
ICCV-201209	40.21 ^a–e^	32.12 ^a–d^	8.09	14.36 ^a–f^	11.27 ^a–g^	3.09	31.56 ^ab^	34.70 ^ab^	3.14	57.47 ^b–r^	50.47 ^a–o^	7.00
ICCV-201113	51.93 ^h–u^	47.83 ^h–t^	4.10	13.48 ^abc^	10.47 ^a–g^	3.01	48.07 ^k–I^	52.17 ^g–y^	4.10	51.98 ^a–f^	43.36 ^a–j^	8.62
ICCV-201118	60.30 ^s–V^	55.69 ^s–y^	4.61	34.09 ^j–l^	30.14 ^e–g^	3.95	39.70 ^b–m^	44.31 ^a–j^	4.61	54.34 ^a–k^	44.23 ^a–k^	10.10
ICCV-201212	58.16 ^p–V^	53.39 ^o–x^	4.77	14.7 ^a–i^	9.65 ^abc^	5.05	41.84 ^b–q^	46.61 ^b–q^	4.77	55.13 ^a–n^	48.35 ^a–n^	6.78
ICCV-201217	57.64 ^o–IV^	50.37 ^j–w^	7.27	15.56 ^a–j^	11.78 ^a–g^	3.78	42.36 ^c–r^	49.63 ^c–v^	7.27	59.45 ^d–t^	55.16 ^a–o^	4.29
ICCV-201102	53.62 ^j–x^	50.48 ^j–w^	3.14	13.53 ^a–d^	11.32 ^a–g^	2.21	46.38 ^h–x^	49.52 ^c–u^	3.14	65.17 ^o–I^	61.87 ^e–o^	3.30
ICCV-201218	49.86 ^e–r^	43.58 ^d–q^	6.28	14.53 ^a–h^	10.2 ^a–f^	4.33	50.14 ^o–IV^	56.42 ^j–z^	6.28	64.61 ^o–I^	60.21 ^c–o^	4.40
ICCV-201108	55.33 ^m–I^	48.21 ^h–t^	7.12	14.25 ^a–f^	13.62 ^a–g^	0.63	44.67 ^e–s^	51.79 ^g–y^	7.12	51.04 ^a–e^	42.33 ^a–h^	8.71
CHAFFA	58.88 ^q–V^	53.60 ^p–y^	5.28	14.36 ^a–f^	12.62 ^a–g^	1.74	41.12 ^b–p^	46.40 ^b–o^	5.28	63.56 ^n–z^	60.24 ^c–o^	3.32
JG-24	40.34 ^a–f^	35.53 ^a–f^	4.81	13.42 ^ab^	11.24 ^a–g^	2.18	59.66 ^III–VI^	64.47 ^x–I^	4.81	67.31 ^t–I^	63.24 ^g–o^	4.07
ICCV-201114	48.45 ^d–p^	41.07 ^d–m^	7.38	14.42 ^a–g^	10.24 ^a–g^	4.18	51.55 ^q–V^	58.93 ^o–I^	7.38	60.54 ^f–u^	55.17 ^a–o^	5.37
ICCV-201107	54.91 ^m–z^	51.53 ^m–w^	3.39	13.53 ^a–d^	11.26 ^a–g^	2.27	45.09 ^f–t^	48.47 ^c–t^	3.39	61.27 ^i–w^	56.17 ^a–o^	5.10
SAGL 22-101	48.22 ^d–o^	41.77 ^d–n^	6.46	14.48 ^a–g^	13.25 ^a–g^	1.23	51.78 ^r–V^	58.23 ^m–I^	6.46	67.38 ^t–I^	64.47 ^i–o^	2.91
SAGL 22-102	68.32 ^V^	63.21 ^x–y^	5.11	13.42 ^abc^	9.75 ^abc^	3.67	43.48 ^ab^	47.75 ^b–s^	4.28	59.28 ^d–t^	47.30 ^a–m^	11.98
SAGL 22-103	64.36 ^y–V^	60.49 ^v–y^	3.86	29.61 ^c–l^	25.31 ^a–g^	4.3	35.64 ^a–g^	39.51 ^a–g^	3.86	56.39 ^a–p^	46.33 ^a–l^	10.06
SAGL 22-104	49.73 ^d–q^	44.21 ^e–r^	5.51	26.33 ^a–l^	23.58 ^a–g^	2.75	50.27 ^p–V^	55.79 ^i–z^	5.51	52.18 ^a–f^	45.27 ^a–k^	6.91
SAGL 22-105	60.47 ^s–V^	53.37 ^o–x^	7.10	16.5 ^a–k^	12.34 ^a–g^	4.16	39.53 ^b–m^	46.63 ^b–q^	7.10	68.48 ^u–I^	63.37 ^g–o^	5.11
SAGL 22-106	42.77 ^a–i^	40.65 ^d–l^	2.12	13.72 ^a–f^	11.37 ^a–g^	2.35	57.23 ^z–VI^	59.35 ^r–I^	2.12	52.49 ^a–h^	45.24 ^a–k^	7.26
SAGL 22-107	50.72 ^f–s^	45.32 ^f–s^	5.40	14.39 ^a–g^	12.42 ^a–g^	1.97	49.28 ^m–III^	54.68 ^i–z^	5.40	54.51 ^a–m^	47.32 ^a–m^	7.19
SAGL 22-108	52.13 ^h–v^	45.28 ^f–s^	6.84	25.78 ^a–l^	21.42 ^a–g^	4.36	47.87 ^j–I^	54.72 ^i–z^	6.84	71.52 ^x–I^	68.34 ^l–o^	3.18
SAGL 22-109	60.81 ^t–V^	55.30 ^s–y^	5.51	15.22 ^a–j^	14.26 ^a–g^	0.96	40.12 ^b–n^	44.70 ^a–j^	4.58	63.35 ^m–y^	59.29 ^b–o^	4.06
SAGL 22-111	42.95 ^b–i^	35.33 ^a–f^	7.62	15.05 ^a–i^	13.25 ^a–g^	1.8	23.98 ^a^	30.58 ^a^	6.60	50.70 ^a–d^	40.33 ^a–f^	10.38
SAGL 22-112	60.98 ^u–V^	54.36 ^q–y^	6.62	15.22 ^a–j^	13.98 ^a–g^	1.24	39.02 ^b–k^	45.64 ^a–l^	6.62	56.55 ^a–q^	50.33	6.22
SAGL 22-113	59.68 ^r–V^	56.39 ^t–y^	3.29	14.5 ^a–g^	10.32 ^a–g^	4.18	40.32 ^b–o^	43.61 ^a–i^	3.29	52.50 ^a–i^	44.32 ^a–k^	8.18
SAGL 22-114	54.43 ^l–y^	49.61 ^i–v^	4.82	14.39 ^a–g^	9.62 ^abc^	4.77	45.57 ^g–u^	50.39 ^d–v^	4.82	63.44 ^n–y^	59.42 ^b–o^	4.02
SAGL 22 -115	43.80 ^b–j^	38.60 ^a–h^	5.21	15.17 ^a–i^	14.75 ^a–g^	0.42	56.20 ^w–VI^	61.40 ^u–I^	5.21	49.40 ^abc^	40.29 ^a–e^	9.10
SAGL-152403	61.93 ^v–V^	54.29 ^q–y^	7.64	32.55 ^i–l^	27.89 ^b–g^	4.66	38.07 ^b–j^	45.71	7.64	49.23 ^ab^	39.47 ^a–d^	9.75
SAGL-152254	44.48 ^c–l^	40.39 ^b–k^	4.09	32.43 ^h–l^	30.12 ^e–g^	6.77	55.52 ^u–VI^	59.61 ^r–I^	4.09	71.25 ^x–I^	68.40 ^l–o^	2.84
SAGL-162370	42.60 ^a–h^	37.84 ^a–h^	4.75	36.89 ^l^	30.67 ^f–g^	6.22	57.40 ^z–VI^	62.16 ^v–I^	4.75	50.61 ^a–d^	43.23 ^a–i^	7.38
SAGL-152210	45.43 ^c–n^	44.52 ^d–m^	0.91	27.05 ^b–l^	25.14 ^a–g^	1.91	60.32 ^IV–VI^	65.74 ^q–I^	5.42	66.47 ^s–I^	55.98 ^a–o^	10.49
SAGL-152273	65.21 ^I–V^	59.28 ^u–y^	5.93	19.28 ^a–l^	16.53 ^a–g^	2.75	34.79 ^a–f^	40.72 ^a–h^	5.93	67.40 ^t–I^	64.38 ^i–o^	3.03
SAGL-152216	32.73 ^ab^	27.53 ^abc^	5.20	24.55 ^a–l^	21.22 ^a–g^	3.33	45.75 ^g–v^	49.64 ^c–v^	3.89	54.27 ^a–k^	43.30 ^a–j^	10.97
RVSSG-64	48.60 ^d–p^	43.44 ^d–p^	5.16	15.27 ^a–j^	11.45 ^a–g^	3.82	51.40 ^q–V^	56.56 ^j–I^	5.16	65.47 ^q–I^	60.14 ^c–o^	5.34
SAGL-162265	50.21 ^e–r^	48.29 ^h–u^	1.92	13.93 ^a–f^	8.62 ^ab^	5.31	49.79 ^n–IV^	51.71 ^f–x^	1.92	61.56 ^j–w^	58.46 ^b–o^	3.10
SAGL-152347	52.20 ^g–t^	50.32 ^h–t^	1.88	17.32 ^a–l^	13.29 ^a–g^	4.03	47.80 ^l–lI^	48.78 ^g–y^	0.97	63.08 ^e–u^	59.44 ^a–o^	3.63
SAGL-162376	45.42 ^c–n^	42.50 ^d–o^	2.92	17.82 ^a–l^	13.44 ^a–g^	4.38	54.58 ^t–VI^	57.50 ^k–I^	2.92	60.42 ^f–u^	55.27 ^a–o^	5.15
SAGL-152314	54.13 ^k–x^	50.59 ^k–w^	3.54	20.27 ^a–l^	15.29 ^a–g^	4.98	45.87 ^h–w^	49.41 ^c–u^	3.54	56.36 ^a–o^	48.41 ^a–n^	7.96
SAGL-162375	36.59 ^abc^	33.28 ^a–e^	3.32	13.71	10.74 ^ab^	2.97	68.32 ^VI^	70.68 ^I^	2.36	55.19 ^a–n^	43.96 ^a–j^	11.24
SAGL-152278	66.80 ^III–IV^	63.44 ^xy^	3.36	25.38 ^a–l^	22.16 ^a–g^	3.22	33.20 ^a–d^	36.56 ^abc^	3.36	60.25 ^f–u^	57.30 ^a–o^	2.95
SAGL-152242	50.97 ^g–t^	45.37 ^f–s^	5.60	14.88 ^a–i^	10.25 ^a–g^	4.63	49.03 ^l–II^	54.63 ^i–z^	5.60	57.31 ^a–r^	55.27 ^a–o^	2.03
SAGL-152238	47.30 ^c–o^	45.56 ^f–s^	1.74	29.88 ^e–l^	25.41 ^a–g^	4.47	52.70 ^s–IV^	54.44 ^i–z^	1.74	65.30 ^p–I^	61.37 ^e–o^	3.93
SAGL-162390	49.99 ^e–r^	41.70 ^d–n^	8.28	14.77 ^a–i^	10.37 ^a–g^	4.4	50.01 ^n–IV^	58.30 ^n–I^	8.28	55.54 ^a–n^	46.32 ^a–l^	9.22
RVSSG-69	66.91 ^IV–V^	61.44 ^w–y^	5.47	13.6 ^a–e^	9.75 ^abc^	3.85	33.09 ^abc^	38.56 ^a–e^	5.47	60.98 ^g–v^	57.36 ^a–o^	3.62
SAGL-152256	56.56 ^n–III^	53.56 ^o–y^	3.00	13.82 ^a–f^	10.34 ^a–g^	3.48	43.44 ^d–s^	46.44 ^b–p^	3.00	52.95 ^a–j^	39.24 ^a–c^	13.72
SAGL-152208	48.34 ^d–p^	45.30 ^f–s^	3.04	14.26 ^a–f^	11.56 ^a–g^	2.7	51.66 ^q–V^	54.70 ^i–z^	3.04	59.42 ^d–t^	54.41 ^a–o^	5.01
SAGL-152303	60.59 ^t–V^	54.53 ^r–y^	6.06	35.65 ^k–l^	32.12 ^g^	3.53	39.41 ^b–l^	45.47 ^a–k^	6.06	62.47 ^k–x^	59.36 ^b–o^	3.11
SAGL-152404	52.83 ^i–x^	41.81 ^d–o^	11.02	14.32 ^a–f^	11.26 ^a–g^	3.06	47.17 ^i–z^	58.19 ^l–I^	11.02	48.37 ^a^	35.30 ^a^	13.07
SAGL-152236	58.70 ^q–R^	53.86 ^p–y^	4.84	16.6 ^a–k^	12.57 ^a–g^	4.03	41.30 ^b–p^	46.14 ^b–n^	4.84	53.40 ^a–j^	43.47 ^a–j^	9.93
SAGL-152252	65.98 ^II–V^	60.56 ^v–y^	5.42	35.62 ^k–l^	30.25 ^f–g^	5.37	34.02 ^a–d^	39.44 ^a–g^	5.42	63.30 ^m–y^	58.20 ^a–o^	5.10
SAGL-152349	57.44 ^o–IV^	55.23 ^s–y^	2.21	29.99 ^f–l^	24.31 ^a–g^	5.68	42.56 ^c–r^	44.77 ^a–j^	2.21	62.83 ^k–y^	60.41 ^c–o^	2.42
SAGL-162389	47.38 ^c–o^	45.24	2.14	32.16	29.86 ^a–g^	2.3	52.62 ^l–II^	54.76	2.14	56.32	42.22 ^a–o^	14.10
SAGL-162371	62.35 ^w–V^	53.97 ^p–y^	8.38	29.82 ^d–l^	22.32 ^a–g^	7.5	37.65 ^b–i^	46.03 ^b–n^	8.38	60.43 ^f–u^	58.29 ^b–o^	2.14
SAGL-152342	54.47 ^l–y^	48.35 ^h–u^	6.12	13.93 ^a–f^	10.24 ^a–g^	3.69	45.53 ^g–u^	51.65 ^e–w^	6.12	54.52 ^a–m^	41.41 ^a–g^	13.10
SAGL-152334	43.66 ^b–j^	40.44 ^c–l^	3.23	15.2 ^a–i^	9.85 ^a–e^	5.35	56.34 ^x–VI^	59.56 ^r–I^	3.23	67.96 ^t–I^	63.40 ^h–o^	4.56
RVSSG-75	53.58 ^j–x^	48.22 ^h–u^	5.36	13.2 ^ab^	8.34 ^ab^	4.86	46.42 ^h–y^	51.78 ^g–y^	5.36	63.14 ^l–y^	60.50 ^c–o^	2.64
JG-14	60.38 ^s–V^	48.32 ^d–m^	12.06	14.7 ^a–i^	10.74 ^a–g^	3.96	42.32 ^b–m^	58.85 ^o–I^	16.53	72.30 ^y–I^	69.30 ^m–o^	3.01
JG-11	59.34 ^q–V^	55.30 ^s–y^	4.04	14.37 ^a–f^	13.25 ^a–g^	1.12	40.66 ^b–p^	44.70 ^a–j^	4.04	74.20 ^I^	73.65 ^o^	0.55
NBeG-47	47.18 ^c–o^	45.37 ^f–s^	1.81	13.98 ^a–f^	8.34 ^ab^	5.64	52.82 ^s–IV^	54.63 ^i–z^	1.81	60.99 ^h–w^	59.64 ^c–o^	1.35
JGG-1	52.45 ^h–w^	51.05 ^l–w^	1.40	13.65 ^a–f^	10.32 ^a–g^	3.33	47.55 ^i–I^	48.95 ^c–u^	1.40	62.50 ^k–x^	58.95 ^b–o^	3.55
RVG-205	56.82 ^n–IV^	52.28 ^n–x^	4.54	18.15 ^a–l^	15.62 ^a–g^	2.53	43.18 ^c–s^	47.72 ^b–r^	4.54	58.26 ^c–s^	52.19 ^a–o^	6.07
RVG-201	63.29 ^x–V^	59.90 ^v–y^	3.39	10.32 ^a^	7.43 ^a^	2.89	36.71 ^b–h^	40.10 ^a–h^	3.39	55.38 ^a–n^	47.34 ^a–m^	8.05
VISHAL	50.74 ^n–II^	47.40 ^g–t^	3.35	21.98 ^a–l^	18.75 ^a–g^	3.23	49.26 ^l–III^	52.60 ^h–y^	3.35	59.30 ^d–t^	54.17 ^a–o^	5.13
JG-63	30.12 ^a^	27.35 ^a^	2.77	15.2 ^a–j^	10.85 ^a–g^	4.35	57.62 ^I–VI^	60.53 ^t–I^	2.91	64.98 ^o–I^	61.24 ^d–o^	3.74
RVSSG-85	65.50 ^q–V^	55.12 ^s–y^	10.38	14.82 ^a–i^	10.35 ^a–g^	4.47	34.50 ^a–e^	45.02 ^i–z^	10.52	70.32 ^w–I^	65.27 ^j–o^	5.04
RVG-210	54.95 ^o–V^	48.37 ^h–u^	6.58	31.87 ^g–l^	29.65 ^c–g^	2.22	45.05 ^f–t^	51.63 ^e–w^	6.58	70.16 ^v–I^	66.24 ^k–o^	3.92
SAGL-161032	57.58 ^u–V^	52.37 ^n–x^	5.21	13.37 ^ab^	10.24 ^a–f^	3.13	42.42 ^c–r^	47.63 ^b–r^	5.21	52.21 ^a–g^	40.14 ^a–e^	12.06
SAGL-163603	39.16 ^abcd^	35.58 ^a–f^	3.58	13.87 ^a–f^	9.75 ^abcd^	4.12	60.84 ^V–VI^	64.42 ^w–I^	3.58	54.43 ^a–l^	41.14 ^a–f^	13.29
SAGL-161018	44.51 ^c–m^	40.60 ^d–l^	3.91	27.54 ^b–l^	22.62 ^a–g^	4.92	55.49 ^u–VI^	59.40 ^r–I^	3.91	53.20 ^a–j^	45.18 ^a–k^	8.02
SAGL-163008	39.95 ^a–e^	35.38 ^a–f^	4.56	15.08 ^a–j^	10.32 ^a–g^	4.76	60.05 ^IV–VI^	64.62 ^y–I^	4.56	50.23 ^abc^	40.21 ^a–e^	10.03
SAGL-161001	54.34 ^k–x^	41.01 ^d–m^	13.33	15.13 ^a–i^	11.24 ^a–g^	3.89	45.66 ^g–u^	58.99 ^p–I^	13.33	61.39 ^j–w^	56.30 ^a–o^	5.09
RVSSG-68	41.05 ^a–g^	36.51 ^a–f^	4.54	16.3 ^a–k^	13.49 ^a–g^	2.81	58.95 ^II–VI^	63.49 ^w–I^	4.54	55.27 ^a–n^	44.21 ^a–k^	11.06
JG-315	65.20 ^z–V^	60.36 ^v–y^	4.84	21.85 ^a–l^	18.79 ^a–g^	3.06	34.80 ^a–f^	39.64 ^a–g^	4.84	65.32 ^q–I^	60.24 ^f–o^	5.08
JG-74	30.21 ^abc^	27.42 ^ab^	2.79	16.74 ^a–f^	12.57 ^a–g^	4.17	60.12 ^IV–VI^	66.58 ^z–I^	6.46	49.01 ^ab^	37.17 ^ab^	11.85
ICC-4958	66.71 ^III–IV^	65.32 ^y^	1.39	19.65 ^a–l^	18.62 ^a–g^	1.03	33.29 ^a–d^	37.79 ^a–d^	4.51	73.54 ^z–I^	70.85 ^n–o^	2.69
Mean	52.55	47.61	4.94	18.92	15.39	3.59	46.44	51.44	5.00	59.63	53.05	6.57
Max	68.32	65.32	13.33	36.89	32.12	7.50	68.32	70.68	16.53	74.20	73.65	14.10
Min	30.12	27.35	0.91	10.32	7.43	0.42	23.98	30.58	0.97	48.37	35.30	0.55
SD	9.50			9.65			9.29			9.06		
SE	0.62			0.63			0.61			0.59		

**Table 2:** Effect of drought on non-enzymatic activity of chickpea genotypes under normal and stressed conditions.

	Chl_a_ (mg g^−1^ FW)			Chl_b_ (mg g^−1^ FW)		
Genotypes	Control	Drought	Reduction	Control	Drought	Reduction
ICCV-201111	0.47 ^a–o^	0.38 ^i–x^	0.09	0.41 ^h–q^	0.36 ^m–x^	0.05
JG-36	0.48 ^b–o^	0.37 ^g–s^	0.11	0.35 ^b–o^	0.31 ^e–x^	0.04
GCP-101	0.47 ^a–o^	0.35 ^d–k^	0.12	0.41 ^h–q^	0.3 ^d–w^	0.11
ICCV-201105	0.46 ^a–n^	0.32 ^bcd^	0.14	0.38 ^c–q^	0.29 ^c–u^	0.09
ICCV-201209	0.45 ^a–m^	0.37 ^h–w^	0.08	0.32 ^a–g^	0.25 ^a–j^	0.07
ICCV-201113	0.48 ^c–o^	0.35 ^d–i^	0.13	0.36 ^b–q^	0.29 ^c–v^	0.07
ICCV-201118	0.5 ^f–o^	0.4 ^u–III^	0.1	0.41 ^h–q^	0.35 ^i–x^	0.06
ICCV-201212	0.45 ^a–j^	0.38 ^m–x^	0.07	0.37 ^b–q^	0.32 ^g–x^	0.05
ICCV-201217	0.48 ^b–o^	0.37 ^h–t^	0.11 ^h–u^	0.28 ^ab^	0.21 ^a–f^	0.07
ICCV-201102	0.53 ^l–o^	0.42 ^II–V^	0.11	0.39 ^d–q^	0.33 ^g–x^	0.06
ICCV-201218	0.43 ^a–g^	0.41 ^y–III^	0.02	0.24 ^a^	0.15 ^a^	0.09
ICCV-201108	0.53 ^l–o^	0.45 ^V–VIII^	0.08	0.43 ^m–q^	0.41 ^w–x^	0.02
CHAFFA	0.47 ^a–o^	0.4 ^x–III^	0.07	0.39 ^d–q^	0.31 ^e–x^	0.08
JG-24	0.48 ^b–o^	0.41 ^z–III^	0.07	0.4 ^g–q^	0.37 ^p–x^	0.03
ICCV-201114	0.45 ^a–l^	0.38 ^l–x^	0.07	0.38 ^c–q^	0.27 ^b–p^	0.11
ICCV-201107	0.52 ^k–o^	0.48 ^VII–XI^	0.04	0.41 ^j–q^	0.37 ^n–x^	0.04
SAGL 22-101	0.49 ^e–o^	0.43 ^III–VI^	0.06	0.42 ^i–q^	0.35 ^i–x^	0.07
SAGL 22-102	0.47 ^a–o^	0.36	0.11	0.35 ^b–q^	0.23 ^a–g^	0.12
SAGL 22-103	0.42 ^a–e^	0.36 ^G–P^	0.06	0.33 ^a–k^	0.28 ^b–r^	0.05
SAGL 22-104	0.45 ^a–k^	0.36 ^j–x^	0.09	0.31 ^a–e^	0.25 ^a–i^	0.06
SAGL 22-105	0.52 ^i–o^	0.47 ^VII–IX^	0.05	0.43 ^n–q^	0.39 ^t–x^	0.04
SAGL 22-106	0.43 ^a–g^	0.36 ^e–n^	0.07	0.35 ^b–q^	0.27	0.08
SAGL 22-107	0.46 ^a–n^	0.37 ^h–w^	0.09	0.34 ^a–n^	0.17 ^ab^	0.17
SAGL 22-108	0.47 ^a–o^	0.37 ^g–s^	0.1	0.32 ^a–h^	0.27 ^p–q^	0.05
SAGL 22-109	0.53 ^l–o^	0.48 ^VII–XI^	0.05	0.41 ^h–q^	0.35 ^j–x^	0.06
SAGL 22-111	0.46 ^a–n^	0.37 ^h–w^	0.09	0.32 ^a–g^	0.21 ^a–f^	0.11
SAGL 22-112	0.48 ^c–o^	0.41 ^z–III^	0.07	0.4 ^f–q^	0.37 ^o–x^	0.03
SAGL 22-113	0.43 ^a–f^	0.37 ^g–t^	0.06	0.39 ^e–q^	0.26 ^a–m^	0.13
SAGL 22-114	0.45 ^a–m^	0.34 ^c–g^	0.11	0.34 ^a–l^	0.28 ^b–r^	0.06
SAGL 22 -115	0.5 ^g–o^	0.47 ^VII–X^	0.03	0.41 ^h–q^	0.39 ^s–x^	0.02
SAGL-152403	0.42 ^a–e^	0.39 ^r–II^	0.03	0.32 ^a–i^	0.18 ^a–c^	0.14
SAGL-152254	0.45 ^a–i^	0.38 ^n–x^	0.07	0.38 ^c–q^	0.35 ^h–x^	0.03
SAGL-162370	0.48 ^b–o^	0.32 ^bcd^	0.16	0.34 ^a–o^	0.21 ^a–f^	0.13
SAGL-152210	0.51 ^h–o^	0.46 ^VI–VIII^	0.05	0.43 ^o–q^	0.39 ^u–x^	0.04
SAGL-152273	0.46 ^a–n^	0.37 ^h–v^	0.09	0.32 ^a–g^	0.2 ^a–d^	0.12
SAGL-152216	0.47 ^a–o^	0.35 ^d–j^	0.12	0.36 ^b–q^	0.31 ^e–x^	0.05
RVSSG-64	0.46 ^a–n^	0.38 ^n–x^	0.08	0.37 ^b–q^	0.3 ^d–w^	0.07
SAGL-162265	0.42 ^a–e^	0.39 ^s–II^	0.03	0.36 ^b–q^	0.31 ^e–w^	0.05
SAGL-152347	0.48 ^a–o^	0.36	0.12	0.32 ^a–o^	0.26 ^c–s^	0.06
SAGL-162376	0.46 ^a–n^	0.4 ^t–II^	0.06	0.31 ^a–e^	0.27 ^b–q^	0.04
SAGL-152314	0.45 ^a–m^	0.39 ^q–I^	0.06	0.33 ^a–j^	0.21 ^a–e^	0.12
SAGL-162375	0.41 ^abc^	0.33 ^b–e^	0.08	0.34 ^a–l^	0.23 ^a–g^	0.11
SAGL-152278	0.49 ^d–o^	0.45 ^IV–VII^	0.04	0.41 ^i–q^	0.36 ^i–x^	0.05
SAGL-152242	0.43 ^a–g^	0.38 ^n–x^	0.05	0.34 ^a–l^	0.26 ^a–k^	0.08
SAGL-152238	0.51 ^i–o^	0.46 ^VI–VIII^	0.05	0.41 ^k–q^	0.37	0.04
SAGL-162390	0.52 ^i–o^	0.49 ^IX–XI^	0.03	0.44 ^p–q^	0.4 ^v–x^	0.04
RVSSG-69	0.41 ^abc^	0.35 ^d–m^	0.06	0.35 ^b–o^	0.27 ^a–o^	0.08
SAGL-152256	0.43 ^a–h^	0.36 ^e–o^	0.07	0.37 ^b–q^	0.31 ^e–w^	0.06
SAGL-152208	0.53 ^m–o^	0.^49 IX–XI^	0.04	0.4 ^f–q^	0.36 ^k–x^	0.04
SAGL-152303	0.43 ^a–f^	0.34 ^c–f^	0.09	0.38 ^c–q^	0.31 ^e–x^	0.07
SAGL-152404	0.48 ^b–o^	0.36 ^h–w^	0.12	0.36 ^b–q^	0.31 ^f–x^	0.05
SAGL-152236	0.45 ^a–m^	0.36 ^g–q^	0.09	0.31 ^a–d^	0.25 ^a–j^	0.06
SAGL-152252	0.52 ^j–o^	0.48 ^VIII–XI^	0.04	0.39 ^d–q^	0.36 ^m–x^	0.03
SAGL-152349	0.43 ^a–g^	0.38 ^III–VI^	0.05	0.31 ^a–e^	0.26 ^a–n^	0.05
SAGL-162389	0.42 ^a–m^	0.35 ^e–n^	0.07	0.37 ^a–o^	0.31	0.06
SAGL-162371	0.54 ^o^	0.51 ^XI^	0.03	0.36 ^b–q^	0.27 ^b–q^	0.09
SAGL-152342	0.48 ^b–o^	0.42 ^I–IV^	0.06	0.32 ^a–f^	0.28 ^c–t^	0.04
SAGL-152334	0.42 ^abc^	0.36 ^e–n^	0.06	0.38 ^c–q^	0.35 ^j–x^	0.03
RVSSG-75	0.41 ^abc^	0.35 ^c–h^	0.06	0.34 ^a–m^	0.26 ^a–m^	0.08
JG-14	0.4 ^a^	0.35 ^d–l^	0.05	0.31 ^a–e^	0.26 ^a–m^	0.05
JG-11	0.51 ^h–o^	0.47 ^VII–IX^	0.04	0.41 ^k–q^	0.37 ^o–x^	0.04
NBeG-47	0.42 ^a–e^	0.36 ^e–n^	0.06	0.39 ^d–q^	0.28 ^b–q^	0.11
JGG-1	0.48 ^c–o^	0.4 ^w–III^	0.08	0.34 ^b–o^	0.3 ^d–w^	0.04
RVG-205	0.45 ^a–k^	0.39 ^s–II^	0.06	0.32 ^a–g^	0.26 ^a–l^	0.06
RVG-201	0.46 ^a–n^	0.39 ^o–z^	0.07	0.35 ^b–o^	0.29 ^d–v^	0.06
VISHAL	0.42 ^a–d^	0.29 ^a^	0.13	0.36 ^b–q^	0.27 ^b–q^	0.09
JG-63	0.47 ^a–o^	0.4 ^v–III^	0.07	0.32 ^a–g^	0.28 ^b–q^	0.04
RVSSG-85	0.52 ^i–o^	0.45 ^V–VIII^	0.07	0.42 ^l–q^	0.39 ^r–x^	0.03
RVG-210	0.43 ^a–h^	0.32 ^abc^	0.11	0.3 ^abc^	0.24 ^a–h^	0.06
SAGL-161032	0.45 ^a–k^	0.39 ^s–II^	0.06	0.34 ^a–l^	0.27 ^b–q^	0.07
SAGL-163603	0.48 ^c–o^	0.33	0.15	0.36 ^b–q^	0.3 ^d–w^	0.06
SAGL-161018	0.49 ^e–o^	0.38 ^k–x^	0.11	0.38 ^c–q^	0.34 ^h–x^	0.04
SAGL-163008	0.42 ^a–d^	0.37 ^h–w^	0.05	0.37 ^b–q^	0.28 ^c–u^	0.09
SAGL-161001	0.43 ^a–g^	0.39 ^p–z^	0.04	0.34 ^a–o^	0.26 ^a–l^	0.08
RVSSG-68	0.42 ^a–d^	0.37	0.05	0.35 ^b–p^	0.29 ^c–u^	0.06
JG-315	0.52 ^i–o^	0.48 ^VII–X^	0.04	0.42 ^l–q^	0.38 ^q–x^	0.04
JG-74	0.41 ^ab^	0.3 ^ab^	0.11	0.35 ^b–p^	0.28 ^b–q^	0.07
ICC-4958	0.53 ^n–o^	0.5 ^X–XI^	0.03	0.44 ^q^	0.42 ^x^	0.02
Mean	0.46	0.39	0.07	0.36	0.29	0.06
Max.	0.54	0.51	0.16	0.44	0.42	0.17
Min.	0.4	0.29	0.02	0.24	0.15	0.02
SD	0.037	0.050		0.041	0.059	
SE	0.23	0.12		0.08	0.06	

**Table 3:** Effect of drought on non-enzymatic activities under normal and stressed conditions.

S.No.	Name of Genotype	MDA (nmol g^−1^ DW)			Sugar (mg g^−1^ Dry Weight)			Phenol (mg Gallic Acid Equivalent g^−1^)			Proline (mg g^−1^ FW)			Protein (%)		
		Normal	Drought	Increase	Normal	Drought	Increase	Normal	Drought	Increase	Normal	Drought	Increase	Normal	Drought	Reduction
1	ICCV-201111	1.56 ^u–z^	1.67 ^d–i^	0.11	29 ^d–h^	37 ^a–e^	8	1.02 ^a–n^	1.05 ^a–k^	0.03	15.26 ^a–d^	40.25 ^a–b^	24.99	22.1 ^a–d^	20.13 ^a–l^	1.97
2	JG-36	1.12 ^ab^	1.15 ^ab^	0.03	26 ^b–d^	35 ^abc^	9	0.87 ^a–j^	1.95 ^w–I^	1.08	17.45 ^a–f^	45.23 ^a–f^	27.78	23.2 ^a–d^	18.52 ^a–k^	4.68
3	GCP-101	1.17 ^a–d^	1.25 ^a–e^	0.08	32 ^i–h^	41 ^e–i^	9	0.86 ^a–i^	1.25 ^c–n^	0.39	13.62 ^ab^	37.12 ^a^	23.5	25.4 ^a–d^	21.03 ^b–l^	4.37
4	ICCV-201105	1.58 ^v–I^	1.65 ^c–i^	0.07	33 ^n–v^	43 ^g–k^	10	0.9 ^a–k^	1.02 ^a–h^	0.12	32.15 ^a–k^	52.12 ^a–n^	19.97	20.2 ^a–d^	15.32 ^a–h^	4.88
5	ICCV-201209	1.55 ^s–y^	1.61 ^b–i^	0.06	30 ^e–m^	45 ^i–o^	15	0.76 ^a–f^	1.82 ^t–I^	1.06	26.38	53.28 ^a–n^	26.89	21.6 ^a–d^	17.42 ^a–k^	4.18
6	ICCV-201113	1.65 ^x–II^	1.71 ^f–i^	0.06	29 ^d–h^	41 ^e–i^	12	0.74 ^ab^	0.85 ^abc^	0.11	15.24 ^a–d^	42.35 ^a–d^	27.11	19.8 ^a–d^	14.2 ^a–e^	5.6
7	ICCV-201118	1.23 ^b–j^	1.31 ^a–i^	0.08	28 ^c–g^	39 ^b–g^	11	0.94 ^a–k^	1.03 ^a–i^	0.09	17.52 ^a–g^	45.75 ^f–g^	28.23	20.7 ^a–d^	15.32 ^a–h^	5.38
8	ICCV-201212	1.42 ^m–s^	1.52 ^a–i^	0.1	26 ^b–d^	34 ^a^	8	0.92 ^a–k^	1.03 ^a–f^	0.11	18.34 ^a–h^	43.25 ^a–e^	24.91	21.3 ^a–d^	16.57 ^a–k^	4.73
9	ICCV-201217	1.62 ^w–II^	1.75 ^g–i^	0.13	25 ^bc^	37 ^a–e^	12	1.12 ^b–o^	1.21 ^b–l^	0.09	15.23 ^abc^	40.28 ^ab^	25.05	18.4 ^abc^	12.95 ^a–d^	5.45
10	ICCV-201102	1.52 ^r–x^	1.58 ^a–i^	0.06	36 ^x–VII^	49 ^p–s^	13	1.62 ^o–w^	1.78 ^r–I^	0.16	13.25 ^a^	41.03 ^a–c^	27.78	16.2 ^ab^	11.27 ^ab^	4.93
11	ICCV-201218	1.22 ^b–i^	1.29 ^a–g^	0.07	29 ^d–i^	36 ^a–d^	7	0.8 ^a–g^	0.89 ^a–e^	0.09	19.62 ^a–i^	50.27 ^a–n^	30.65	15.4 ^a^	10.24 ^a^	5.16
12	ICCV-201108	1.67 ^y–II^	1.7 ^d–i^	0.03	36 ^v–VII^	59 ^w–y^	23	1.75 ^r–w^	1.95 ^w–I^	0.2	35.42 ^e–k^	67.45 ^j–n^	32.03	20.3 ^a–d^	18.32 ^a–k^	1.98
13	CHAFFA	1.23 ^b–j^	1.31 ^a–i^	0.08	31 ^h–p^	46 ^j–q^	15	1.2 ^b–q^	1.3 ^e–p^	0.1	20.35 ^a–j^	46.35 ^a–h^	26	19.3 ^a–d^	14.26 ^a–f^	5.04
14	JG-24	1.59 ^w–II^	1.63 ^c–i^	0.04	32 ^j–s^	52 ^s–v^	20	1.34 ^i–w^	1.57 ^i–w^	0.23	37.4 ^f–k^	60.21 ^c–n^	22.81	23.2 ^a–d^	21.03 ^b–l^	2.17
15	ICCV-201114	1.33 ^f–p^	1.43 ^a–i^	0.1	31 ^h–q^	40 ^c–h^	9	0.98 ^a–m^	1.12 ^a–k^	0.14	20.21 ^a–j^	48.57 ^a–i^	28.36	22.1 ^a–d^	17.45 ^a–k^	4.65
16	ICCV-201107	1.62 ^w–II^	1.65 ^c–i^	0.03	34 ^p–x^	56 ^u–x^	22	0.85 ^a–i^	1.98 ^x–I^	1.13	34.52 ^c–k^	64.29 ^f–n^	29.77	20.3 ^a–d^	19.52 ^a–l^	0.78
17	SAGL 22-101	1.56 ^t–y^	1.59 ^a–i^	0.03	40 ^VIII^	61 ^x–y^	21	1.24 ^b–r^	1.45 ^i–v^	0.21	32.24 ^a–k^	63.58 ^f–n^	31.34	20.1 ^a–d^	18.52 ^a–k^	1.58
18	SAGL 22-102	1.32 ^e–o^	1.4 ^a–i^	0.08	37 ^V–VIII^	50 ^q–t^	13	0.87 ^a–j^	1.96 ^w–I^	1.09	15.42 ^a–d^	43.29 ^a–e^	27.87	18.5 ^a–c^	12.45 ^a–c^	6.05
19	SAGL 22-103	1.32 ^e–o^	1.42 ^a–i^	0.1	32 ^m–u^	48 ^m–s^	16	1.25 ^e–s^	1.34 ^f–q^	0.09	36.29 ^e–k^	57.89 ^b–n^	21.6	21.6 ^a–d^	16.23 ^a–i^	5.37
20	SAGL 22-104	1.02 ^a^	1.1 ^a^	0.08	30 ^g–o^	43 ^g–k^	13	1.67 ^q–w^	1.73 ^q–I^	0.06	35.28 ^d–k^	55.29 ^a–n^	20.01	20.1 ^a–d^	14.26 ^a–f^	5.84
21	SAGL 22-105	1.57 ^v–I^	1.59 ^a–i^	0.02	38 ^VI–VIII^	58 ^w–y^	20	1.85 ^w^	2.03 ^z–I^	0.18	36.85 ^h–k^	65.86 ^h–n^	29.01	22.3 ^a–d^	20.34 ^a–l^	1.96
22	SAGL 22-106	1.23 ^b–i^	1.26 ^a–d^	0.03	30 ^g–o^	45 ^i–p^	15	1.62 ^o–w^	1.76 ^r–I^	0.14	26.54 ^a–k^	40.27 ^a–b^	13.73	24.5 ^a–d^	20.13 ^a–l^	4.37
23	SAGL 22-107	1.46 ^p–v^	1.54 ^a–i^	0.08	25 ^bc^	40 ^d–h^	15	0.88 ^a–j^	0.95 ^a–f^	0.07	33.62 ^b–k^	55.42 ^a–n^	21.8	26.5 ^a–d^	22.62 ^e–l^	3.88
24	SAGL 22-108	1.21 ^b–j^	1.32 ^a–i^	0.11	28 ^c–g^	42 ^f–j^	14	0.85 ^a–i^	1.05 ^a–k^	0.2	24.96 ^a–k^	46.35 ^a–h^	21.39	23.5 ^a–d^	19.22 ^a–l^	4.28
25	SAGL 22-109	1.52 ^q–w^	1.55 ^a–i^	0.03	36 ^v–VII^	59 ^w–x^	23	1.47 ^l–w^	1.79 ^u–I^	0.32	39.42 ^k^	65.42 ^g–n^	26	24.1 ^a–d^	22.37 ^e–l^	1.73
26	SAGL 22-111	1.62 ^w–II^	1.71 ^e–i^	0.09	35 ^t–VI^	49 ^p–s^	14	1.42 ^k–w^	1.64 ^n–z^	0.22	34.25 ^c–k^	58.34 ^b–n^	24.09	20.3 ^a–d^	17.45 ^a–k^	2.85
27	SAGL 22-112	1.59 ^w–II^	1.62 ^b–i^	0.03	37 ^y–VIII^	55 ^t–w^	18	1.75 ^s–w^	1.95 ^w–I^	0.2	35.32 ^e–k^	61.29 ^d–n^	25.97	27.4 ^b–d^	25.62 ^j–l^	1.78
28	SAGL 22-113	1.26 ^b–j^	1.33 ^a–i^	0.07	32 ^k–s^	47 ^k–r^	15	1.32 ^h–u^	1.42 ^h–u^	0.1	30.25 ^a–k^	54.26 ^a–n^	24.01	20.3 ^a–d^	15.42 ^a–h^	4.88
29	SAGL 22-114	1.12 ^abc^	1.19 ^abc^	0.07	25 ^bc^	38 ^a–f^	13	1.62 ^o–w^	1.77 ^s–I^	0.15	25.42 ^a–k^	46.26 ^a–h^	20.84	21.3 ^a–d^	18.52 ^a–k^	2.78
30	SAGL 22 -115	1.63 ^w–II^	1.66 ^c–i^	0.03	30 ^g–o^	52 ^s–v^	22	1.52 ^m–w^	1.86 ^v–I^	0.34	30.42 ^a–k^	61.23 ^d–n^	30.81	24.9 ^a–d^	22.32 ^d–l^	2.58
31	SAGL-152403	1.25 ^d–l^	1.42 ^a–i^	0.17	32 ^o–w^	40 ^d–h^	8	0.98 ^a–l^	1.05 ^a–k^	0.07	31.26 ^a–k^	54.37 ^a–n^	23.11	22.4 ^a–d^	18.52 ^a–k^	3.88
32	SAGL-152254	1.62 ^w–II^	1.7 ^d–i^	0.08	34 ^r–IV^	50 ^q–t^	16	0.85 ^a–i^	1 ^a–f^	0.15	28.62 ^a–k^	57.42 ^a–n^	28.8	21.6 ^a–d^	16.75 ^a–k^	4.85
33	SAGL-162370	1.22 ^b–h^	1.29 ^a–g^	0.07	35 ^u–VI^	42 ^f–j^	7	0.84 ^a–h^	0.95 ^a–f^	0.11	34.36 ^c–k^	57.43 ^a–n^	23.07	24.6 ^a–d^	21.02 ^a–l^	3.58
34	SAGL-152210	1.58 ^v–I^	1.61 ^b–i^	0.03	39 ^VII–VIII^	60 ^w–y^	21	1.65 ^p–w^	1.92 ^w–I^	0.27	37.45 ^i–k^	69.85 ^l–n^	32.4	30.3 ^d^	28.45 ^l^	1.85
35	SAGL-152273	1.34 ^g–p^	1.43 ^a–i^	0.09	32 ^l–u^	48 ^o–s^	16	0.75 ^a–e^	0.85 ^a–d^	0.1	25.42 ^a–k^	56.32 ^a–n^	30.9	24.1 ^a–d^	20.34 ^a–l^	3.76
36	SAGL-152216	1.25 ^c–k^	1.32 ^a–i^	0.07	37 ^II–VIII^	50 ^q–t^	13	0.88 ^a–j^	1.02 ^a–f^	0.14	28.61 ^a–k^	59.82 ^b–n^	31.21	22.1 ^a–d^	19.56 ^a–l^	2.54
37	RVSSG-64	1.28 ^d–l^	1.36 ^a–i^	0.08	34 ^p–z^	48 ^l–s^	14	0.85 ^a–i^	0.98 ^a–g^	0.13	17.34 ^a–e^	48.27 ^a–j^	30.93	21.3 ^a–d^	16.53 ^a–j^	4.77
38	SAGL-162265	1.25 ^b–j^	1.31 ^a–i^	0.06	29 ^d–l^	43 ^g–l^	14	0.86 ^a–j^	1.01 ^a–j^	0.15	26.34 ^a–k^	54.75 ^a–n^	28.41	26.5 ^a–d^	23.49 ^e–l^	3.01
39	SAGL-152347	1.34 ^l–q^	1.39 ^a–i^	0.05	30 ^h–r^	42 ^h–o^	12	0.95 ^a–g^	1.06 ^a–e^	0.11	16.95 ^a–k^	39.85 ^a–j^	22.9	30.4 d	26.47 ^a–l^	3.93
40	SAGL-162376	1.26 ^c–k^	1.35 ^a–i^	0.09	25 ^bc^	40 ^d–h^	15	1.02 ^a–n^	1.1 ^a–k^	0.08	31.42 ^a–k^	54.73 ^a–n^	23.31	24.2 ^a–d^	21.32 ^d–l^	2.88
41	SAGL-152314	1.22 ^b–g^	1.28 ^a–f^	0.06	27 ^b–e^	39 ^b–g^	12	1.32 ^h–u^	1.45 ^j–v^	0.13	24.32 ^a–k^	56.35 ^a–n^	32.03	20.6 ^a–d^	16.25 ^a–i^	4.35
42	SAGL-162375	1.42 ^n–s^	1.51 ^a–i^	0.09	32 ^m–u^	42 ^f–j^	10	1.27 ^g–t^	1.39 ^g–t^	0.12	26.95 ^a–k^	59.62 ^b–n^	32.67	23.5 ^a–d^	19.52 ^b–l^	3.98
43	SAGL-152278	1.62 ^w–II^	1.7 ^d–i^	0.08	37 ^z–VIII^	58 ^w–y^	21	1.86 ^w^	2.02 ^z–I^	0.16	34.62 ^d–k^	62.34 ^e–n^	27.72	26.5 ^a–d^	24.98 ^b–l^	1.52
44	SAGL-152242	1.24 ^b–j^	1.32 ^a–i^	0.08	24 ^b^	47 ^n–s^	23	1.52 ^n–w^	1.67 ^o–I^	0.15	30.12 ^a–k^	52.16 ^a–n^	22.04	18.6 ^abc^	14.35 ^a–g^	4.25
45	SAGL-152238	1.66 ^y–II^	1.73 ^f–i^	0.07	34 ^q–III^	56 ^u–x^	22	1.82 ^u–w^	2.05 ^I^	0.23	36.52 ^g–k^	67.45 ^j–n^	30.93	21.6 ^a–d^	19.68 ^a–l^	1.92
46	SAGL-162390	1.7 ^II^	1.76 ^i^	0.06	37 ^IV–VIII^	59 ^b–g^	22	1.79 ^t–w^	1.99 ^x–I^	0.2	38.25 ^i–k^	69.34 ^j–n^	31.09	23.5 ^a–d^	22.32 ^d–l^	1.18
47	RVSSG-69	1.24 ^b–j^	1.32 ^a–i^	0.08	24 ^b^	39 ^b–g^	15	1.32 ^h–v^	1.42 ^h–u^	0.1	26.34 ^a–k^	49.61 ^a–n^	23.27	22.3 ^a–d^	18.52 ^a–k^	3.78
48	SAGL-152256	1.33 ^h–p^	1.41 ^a–i^	0.08	20 ^a^	36 ^a–d^	16	1.42 ^k–w^	1.58 ^i–x^	0.16	21.03 ^a–k^	42.45 ^a–d^	21.42	21.4 ^a–d^	19.65 ^a–l^	1.75
49	SAGL-152208	1.42 ^n–t^	1.5 ^a–i^	0.08	34 ^p–II^	56 ^u–x^	22	1.62 ^o–w^	1.92 ^w–I^	0.3	34.26 ^c–k^	66.31 ^i–n^	32.05	23.4 ^a–d^	21.85 ^c–l^	1.55
50	SAGL-152303	1.32 ^e–o^	1.42 ^a–i^	0.1	28 ^c–g^	40 ^d–h^	12	0.75 ^abc^	0.84 ^ab^	0.09	30.12 ^a–k^	52.31 ^a–n^	22.19	25.3 ^a–d^	22.12 ^d–l^	3.18
51	SAGL-152404	1.24 ^b–j^	1.29 ^a–h^	0.05	37 ^III–VIII^	46 ^j–q^	9	1.15 ^b–p^	1.24 ^b–l^	0.09	34.52 ^c–k^	58.31 ^b–n^	23.79	27.4 ^a–d^	23.26 ^e–l^	4.14
52	SAGL-152236	1.41 ^m–r^	1.5 ^a–i^	0.09	24 ^b^	34 ^ab^	10	0.75 ^a–d^	0.88 ^a–d^	0.13	34.52 ^c–k^	59.61 ^b–n^	25.09	23.4 ^a–d^	19.75 ^a–l^	3.65
53	SAGL-152252	1.69 ^I–II^	1.^76 h–i^	0.07	36 ^w–VII^	60 ^w–y^	24	1.68 ^q–w^	1.89 ^w–I^	0.21	39.85 ^k^	70.12 ^m–n^	30.27	25.6 ^a–d^	24.32 ^h–l^	1.28
54	SAGL-152349	1.26 ^d–l^	1.35 ^a–i^	0.09	29 ^d–h^	42 ^f–j^	13	0.75 ^a–d^	0.87 ^a–d^	0.12	31.25 ^a–k^	53.26 ^a–n^	22.01	23.5 ^a–d^	20.32 ^a–l^	3.18
55	SAGL-162389	1.42	1.52 ^a–i^	0.1	32	45	13	0.62 ^ab^	0.76	0.14	34.25 ^a–k^	57.26 ^a–n^	23.01	21.4 ^a–d^	15.62 ^a–l^	5.78
56	SAGL-162371	1.62 ^w–II^	1.74 ^g–i^	0.12	25 ^bc^	38 ^a–f^	13	1.26 ^f–s^	1.38 ^g–s^	0.12	30.25 ^a–k^	55.62 ^a–n^	25.37	24.7 ^a–d^	20.18 ^a–l^	4.52
57	SAGL-152342	1.32 ^e–o^	1.38 ^a–i^	0.06	29 ^d–k^	45 ^i–p^	16	1.52 ^n–w^	1.67 ^n–z^	0.15	24.62 ^a–k^	49.34 ^a–j^	24.72	27.5 ^b–d^	24.16 ^h–l^	3.34
58	SAGL-152334	1.29 ^d–n^	1.37 ^a–i^	0.08	34 ^s–V^	47 ^k–r^	13	0.75 ^a–e^	0.87 ^a–d^	0.12	20.16 ^a–i^	42.31 ^a–d^	22.15	29.6 ^cd^	25.62 ^j–l^	3.98
59	RVSSG-75	1.27 ^d–l^	1.32 ^a–i^	0.05	30 ^f–o^	43 ^g–k^	13	1.42 ^k–w^	1.65 ^m–z^	0.23	27.16 ^a–k^	50.12 ^a–n^	22.96	22.3 ^a–d^	20.32 ^a–l^	1.98
60	JG-14	1.34 ^f–p^	1.42 ^a–i^	0.08	36 ^v–VII^	49 ^p–s^	13	1.67 ^q–w^	1.85 ^v–I^	0.18	30.12 ^a–k^	52.31 ^a–n^	22.19	25.6 ^a–d^	21.37 ^c–l^	4.23
61	JG-11	1.58 ^v–I^	1.68 ^d–i^	0.1	39 ^VII–VIII^	62 ^y^	23	1.8 ^u–w^	2.03 ^z–I^	0.23	35.48 ^e–k^	67.45 ^j–n^	31.97	27.8 ^cd^	25.42 ^i–l^	2.38
62	NBeG-47	1.29 ^d–m^	1.37 ^a–i^	0.08	29 ^d–j^	41 ^e–i^	12	1.37 ^j–w^	1.62 ^i–y^	0.25	26.86 ^a–k^	54.27 ^a–n^	27.41	23.1 ^a–d^	19.36 ^a–l^	3.74
63	JGG-1	1.34 ^i–p^	1.42 ^a–i^	0.08	30 ^e–n^	43 ^g–k^	13	1.52 ^n–w^	1.65 ^m–z^	0.13	29.75 ^a–k^	50.28 ^a–n^	20.53	24.2 ^a–d^	20.87 ^b–l^	3.33
64	RVG-205	1.52 ^q–w^	1.59 ^a–i^	0.07	34 ^p–y^	50 ^q–t^	16	1.34 ^h–w^	1.57 ^i–w^	0.23	34.56 ^c–k^	56.75 ^a–n^	22.19	23.6 ^a–d^	20.31 ^a–l^	3.29
65	RVG-201	1.34 ^f–p^	1.41 ^a–i^	0.07	26 ^b–d^	37 ^a–e^	11	1.62 ^o–w^	1.84 ^u–I^	0.22	37.85 ^i–k^	59.35 ^b–n^	21.5	25.4 ^a–d^	20.42 ^b–l^	4.98
66	VISHAL	1.42 ^o–u^	1.53 ^a–i^	0.11	27 ^b–f^	41 ^e–i^	14	1.25 ^c–s^	1.37 ^f–r^	0.12	34.52 ^c–k^	55.85 ^a–n^	21.33	26.3 ^a–d^	22.68 ^e–l^	3.62
67	JG-63	1.59 ^v–II^	1.65 ^c–i^	0.06	30 ^e–m^	43 ^g–n^	13	1.24 ^b–q^	1.39 ^g–s^	0.15	29.62 ^a–k^	50.12 ^a–l^	20.5	24.2 ^a–d^	21.43 ^c–l^	2.77
68	RVSSG-85	1.62 ^w–II^	1.7 ^d–i^	0.08	34 ^p–I^	57 ^w–y^	23	1.59 ^o–w^	1.86 ^v–I^	0.27	36.57 ^h–k^	63.25 ^f–n^	26.68	26.5 ^a–d^	24.35 ^h–l^	2.15
69	RVG-210	1.22 ^b–f^	1.32 ^a–i^	0.1	36 ^v–VII^	50 ^q–t^	14	1.12 ^b–o^	1.25 ^c–n^	0.13	28.45 ^a–k^	59.64 ^b–n^	31.19	30.1 ^d^	26.52 ^i–l^	3.58
70	SAGL-161032	1.35 ^j–p^	1.44 ^a–i^	0.09	34 ^s–V^	51 ^r–u^	17	0.98 ^a–l^	1.1 ^a–k^	0.12	26.34 ^a–k^	56.35 ^a–n^	30.01	24.2 ^a–d^	21.42 ^c–l^	2.78
71	SAGL-163603	1.45 ^p–v^	1.54 ^a–i^	0.09	28 ^c–g^	43 ^g–m^	15	0.57 ^a^	0.74 ^a^	0.17	39.21 ^j–k^	59.12 ^b–n^	19.91	20.1 ^a–d^	15.42 ^a–h^	4.68
72	SAGL-161018	1.38 ^k–p^	1.48 ^a–i^	0.1	24 ^b^	40 ^d–h^	16	1.25 ^d–s^	1.42 ^h–u^	0.17	26.52 ^a–k^	58.42 ^b–n^	31.9	27.4 ^b–d^	23.52 ^f–l^	3.88
73	SAGL-163008	1.27 ^d–l^	1.36 ^a–i^	0.09	30 ^g–o^	42 ^f–j^	12	1.12 ^b–o^	1.26 ^d–o^	0.14	22.32 ^a–k^	50.32 ^a–n^	28	24.3 ^a–d^	21.34 ^c–l^	2.96
74	SAGL-161001	1.41 ^m–r^	1.48 ^a–i^	0.07	32 ^m–u^	46 ^j–q^	14	1.62 ^o–w^	1.72 ^p–I^	0.1	17.92 ^a–h^	40.12 ^a–b^	22.2	25.3 ^a–d^	20.19 ^a–l^	5.11
75	RVSSG-68	1.22 ^b–e^	1.31 ^a–i^	0.09	26 ^b–d^	40 ^c–h^	14	0.85 ^a–i^	1 ^a–f^	0.15	26.85 ^a–k^	49.37 ^a–j^	22.52	22.4 ^a–d^	16.35 ^a–j^	6.05
76	JG-315	1.69 ^I–II^	1.72 ^f–i^	0.03	37 ^I–VIII^	59 ^w–y^	22	1.84 ^v–w^	2.03 ^z–I^	0.19	35.45 ^e–k^	67.42 ^i–n^	31.97	27.6 ^cd^	25.89 ^k–l^	1.71
77	JG-74	1.26 ^d–k^	1.34 ^a–i^	0.08	30 ^e–m^	46 ^j–q^	16	1.32 ^h–v^	1.45 ^k–v^	0.13	26.85 ^a–k^	50.12 ^a–m^	23.27	24.3 ^a–d^	19.56 ^a–l^	4.74
78	ICC-4958	1.68 ^z–II^	1.71 ^f–i^	0.03	38 ^IV–VIII^	61 ^x–y^	23	1.82 ^v–w^	2.01 ^y–I^	0.19	39.85 ^k^	70.25 ^n^	30.4	25.6 ^a–d^	23.62 ^g–l^	1.98
	Mean	1.40	1.47	0.07	31.3	46.32	15.01	1.23	1.43	0.20	28.59	54.48	25.89	23.36	19.7	3.56
	Max.	1.7	1.76	0.17	40	62	24	1.86	2.05	1.13	39.85	70.25	32.67	30.4	28.45	6.05
	Min.	1.02	1.1	0.02	20	34	7	0.57	0.74	0.03	13.25	37.12	13.73	15.4	10.24	0.78
	SD	0.172	0.167		4.45	7.57		0.367	0.404		7.323	8.493		3.078	3.756	
	SE	0.02	0.02		0.31	0.50		0.03	0.03		0.48	0.55		0.26	0.28	

**Table 4:** Effect of drought on enzymatic activities under normal and stressed conditions.

S. No.	Name of Genotype	DPPH (%)			H_2_O_2_ (mmol g^−1^ FW)			CAT (mg protein^−1^)			APX (µmol min^−1^g^−1^ FW)			SOD (mg protein^−1^)			POD (mg protein^−1^)		
		Normal	Drought	Increase	Normal	Drought	Increase	Normal	Drought	Increase	Normal	Drought	Increase	Normal	Drought	Increase	Normal	Drought	Increase
1	ICCV-201111	5.35 ^a–j^	5.56 ^a–h^	0.21	2.21 ^a–h^	4.25 ^x–IV^	2.04	19.38 ^f–p^	22.85 ^g–p^	3.47	2.65 ^a^	4.25 ^a^	1.6	147 ^o–t^	152 ^qr^	5	1.4 ^iq^	1.45 ^h–l^	0.05
2	JG-36	6.85 ^f–t^	6.99 ^b–k^	0.14	3.57 ^c–n^	4.16 ^s–z^	0.59	35.02 ^II^	40.26 ^z–II^	5.24	2.98 ^b^	5.2 ^e–h^	2.22	159 ^w–IV^	167 ^wx^	8	2 ^y–II^	2.06 ^u–w^	0.06
3	GCP-101	6.34 ^c–t^	6.65 ^a–k^	0.31	3.42 ^a–n^	4.15 ^s–z^	0.73	16.52 ^d–m^	20.33 ^e–n^	3.81	3.62 ^e–h^	5.21 ^e–h^	1.59	150 ^p–v^	154 ^pq^	4	1.6 ^qs^	1.65 ^o–q^	0.05
4	ICCV-201105	5.03 ^a–f^	5.24 ^a–e^	0.21	3.75 ^e–n^	4.32 ^z–VI^	0.57	14.25 ^a–j^	17.45 ^a–k^	3.2	4.12 ^k–m^	7.52 ^s–u^	3.4	143 ^l–q^	150 ^n^	7	1.59 ^pr^	1.65 ^n–q^	0.06
5	ICCV-201209	6.32 ^b–t^	6.55 ^a–k^	0.23	2.03 ^a–f^	3.95 ^m–s^	1.92	20.12 ^h–s^	23.62 ^g–r^	3.5	3.24 ^bc^	5.32 ^g–i^	2.08	150 ^p–w^	156 ^rs^	6	1.37 ^fm^	1.4 ^f–k^	0.03
6	ICCV-201113	4.02 ^a^	4.32 ^a^	0.3	3.65 ^d–n^	3.89 ^k–p^	0.24	21.34 ^k–v^	25.62 ^k–t^	4.28	2.98 ^b^	4.85 ^c–d^	1.87	161 ^I–VII^	168 ^x^	7	1.35 ^el^	1.38 ^e–k^	0.03
7	ICCV-201118	5.21 ^a–g^	5.4 ^a–g^	0.19	3.52 ^b–n^	3.76 ^a^	0.24	20.14 ^h–s^	24.25 ^h–s^	4.11	3.64 ^e–h^	5.24 ^g–j^	1.6	152 ^q–z^	158 ^t^	6	1.45 ^jq^	1.5 ^k–o^	0.05
8	ICCV-201212	6.42 ^d–t^	6.69 ^a–k^	0.27	3.12 ^a–n^	4.11 ^q–x^	0.99	26.54 ^s–I^	30.12 ^o–x^	3.58	4.25 ^l–n^	6.54 ^p–r^	2.29	135 ^g–l^	145 ^m^	10	1.4 ^ho^	1.49 ^j–n^	0.09
9	ICCV-201217	6.32 ^b–t^	6.61 ^a–k^	0.29	3.26 ^a–n^	4.37 ^II–VIII^	1.11	23.51 ^l–x^	27.36 ^l–u^	3.85	3.58 ^e–g^	4.98 ^d–f^	1.4	130 ^d–h^	136 ^h^	6	1.3 ^ch^	1.35 ^c–h^	0.05
10	ICCV-201102	5.03 ^a–f^	5.24 ^a–e^	0.21	2.12 ^a–g^	3.25 ^cd^	1.13	12.52 ^a–g^	15.66 ^a–h^	3.14	4.67 ^p–s^	5.64 ^i–m^	0.97	145 ^m–r^	153 ^op^	8	1.19 ^bc^	1.25 ^bcd^	0.06
11	ICCV-201218	5.98 ^a–r^	6.33 ^a–k^	0.35	3.89 ^f–n^	4.06 ^o–w^	0.17	7.42 ^a^	9.69 ^ab^	2.27	4.87 ^u–v^	6.24 ^o–q^	1.37	128 ^b–g^	135 ^gh^	7	1.39 ^go^	1.42 ^h–k^	0.03
12	ICCV-201108	7.52 ^l–t^	7.6 ^e–k^	0.08	4.21 ^g–n^	4.57 ^VII–X^	0.36	27.43 ^u–II^	36.52 ^v–II^	9.09	5.67 ^III–IV^	7.45 ^s–t^	1.78	161 ^I–VII^	177 ^II^	16	1.85 ^wz^	1.98 ^t–u^	0.13
13	CHAFFA	6.34 ^c–t^	6.58 ^a–k^	0.24	3.54 ^c–n^	4.17 ^t–I^	0.63	7.42 ^a^	10.52 ^abcd^	3.1	4.86 ^u–v^	5.42 ^g–k^	0.56	132 ^f–k^	138 ^i^	6	1.2 ^bc^	1.25 ^b–d^	0.05
14	JG-24	6.85 ^f–t^	6.95 ^b–k^	0.1	4.38 ^a–n^	4.42 ^IV–VII^	0.04	21.03 ^j–u^	32.62 ^s–z^	11.59	5.98 ^IV–VI^	8.45 ^u^	2.47	161 ^I–VII^	178 ^II^	17	1.55 ^or^	1.73 ^q–s^	0.18
15	ICCV-201114	6.52 ^d–t^	6.74 ^a–k^	0.22	3.62 ^d–n^	4.2 ^t–II^	0.58	13.62 ^a–i^	16.54 ^a–j^	2.92	4.32 ^m–p^	5.23 ^e–h^	0.91	160 ^z–IV^	165 ^v^	5	0.98 ^a^	1.02 ^a^	0.04
16	ICCV-201107	7.68 ^n–t^	7.72 ^f–k^	0.04	4.37 ^f–n^	4.49 ^VI–IX^	0.12	24.31 ^n–z^	35.42 ^u–II^	11.11	6.12 ^VI^	9.62 ^w–x^	3.5	156 ^t–II^	174 ^I^	18	1.85 ^wz^	2.03 ^u–v^	0.18
17	SAGL 22-101	7.98 ^q–t^	8.06 ^i–k^	0.08	4.2 ^f–n^	4.31 ^z–VI^	0.11	26.32 ^q–I^	37.41 ^x–II^	11.09	5.64 ^III–IV^	8.52 ^u^	2.88	165 ^III–VIII^	179 ^II–III^	14	2.09 ^II^	2.17 ^v–w^	0.08
18	SAGL 22-102	6.57 ^d–t^	6.78 ^a–k^	0.21	2.12 ^a–g^	4.23 ^u–IV^	2.11	7.51 ^a–b^	9.23 ^a^	1.72	3.84 ^g–k^	5.26 ^e–h^	1.42	150 ^q–x^	155 ^qr^	5	1.45 ^lq^	1.49 ^i–m^	0.04
19	SAGL 22-103	5.23 ^a–h^	5.5 ^a–h^	0.27	2.54 ^a–n^	3.58 ^e–h^	1.04	15.42 ^c–k^	20.13 ^e–n^	4.71	4.62 ^q–u^	6.34 ^g–j^	1.72	137 ^g–m^	142 ^kl^	5	1.42 ^hp^	1.46 ^h–l^	0.04
20	SAGL 22-104	6.35 ^c–t^	6.65 ^a–k^	0.3	3.62 ^d–n^	4.25 ^x–IV^	0.63	19.85 ^h–r^	25.11 ^j–s^	5.26	3.42 ^c–e^	5.28 ^f–h^	1.86	140 ^j–o^	149 ^n^	9	1.3 ^ck^	1.35 ^c–h^	0.05
21	SAGL 22-105	8.32 ^t^	8.41 ^k^	0.09	4.32 ^a–n^	4.46 ^V–IX^	0.14	29.54 ^z–II^	44.46 ^II^	14.92	5.2 ^w–I^	9.52 ^w^	4.32	175 ^IX–XI^	192 ^VII^	17	2.01 ^z–II^	2.15 ^v–w^	0.14
22	SAGL 22-106	6.34 ^c–t^	6.48 ^a–k^	0.14	2.54 ^a–n^	3.9 ^l–r^	1.36	23.61 ^m–y^	27.42 ^m–u^	3.81	4.32 ^m–p^	6.45 ^o–r^	2.13	134 ^g–k^	140 ^j^	6	1.2 ^bd^	1.23 ^bc^	0.03
23	SAGL 22-107	5.12 ^a–f^	5.32 ^a–g^	0.2	1.26 ^ab^	3.52 ^e–f^	2.26	21.03 ^j–u^	26.34 ^l–t^	5.31	4.87 ^u–v^	7.45 ^s–t^	2.58	123 ^a–f^	125 ^d^	2	1.59 ^pr^	1.65 ^o–q^	0.06
24	SAGL 22-108	5.84 ^a–p^	6.1 ^a–k^	0.26	2.54 ^a–n^	4.39 ^III–VII^	1.85	30.12 ^x–II^	34.12 ^t–I^	4	3.62 ^e–h^	5.29 ^e–h^	1.67	160 ^y–VI^	164 ^uv^	4	1.25 ^bf^	1.29 ^c–h^	0.04
25	SAGL 22-109	7.67 ^m–t^	7.74 ^f–k^	0.07	4.29 ^h–n^	4.42 ^IV–VII^	0.13	27.42 ^u–II^	36.52 ^w–II^	9.1	5.32 ^x–II^	10.24 ^y^	4.92	137 ^g–n^	150 ^n^	13	1.85 ^vz^	1.98 ^t–u^	0.13
26	SAGL 22-111	4.32 ^abc^	4.6 ^ab^	0.28	2.56 ^a–n^	4.32 ^z–VI^	1.76	20.12 ^h–s^	23.62 ^g–q^	3.5	3.21 ^bc^	6.42 ^o–q^	3.21	150 ^p–x^	154 pq	4	1.29 ^ch^	1.34 ^c–h^	0.05
27	SAGL 22-112	7.45 ^k–t^	7.52 ^e–k^	0.07	4.59 ^l–n^	4.74 ^X–XII^	0.15	23.25 ^l–v^	40.32 ^z–II^	17.07	5.75 ^IV–V^	11.03 ^z^	5.28	175 ^IX–XI^	185 ^IV^	10	1.99 ^y–II^	2.12 ^u–w^	0.13
28	SAGL 22-113	5.62 ^a–m^	5.75 ^a–j^	0.13	3.45 ^a–n^	4.15 ^s–z^	0.7	18.56 ^e–o^	22.34 ^g–o^	3.78	4.38 ^m–q^	6.34 ^o–q^	1.96	151 ^q–y^	156 ^rs^	5	1.35 ^el^	1.36 ^d–j^	0.01
29	SAGL 22-114	5.37 ^a–j^	5.64 ^a–i^	0.27	2.75 ^a–n^	4.17 ^t–l^	1.42	15.42 ^c–k^	19.65 ^d–m^	4.23	4.61 ^q–u^	5.98 ^m–o^	1.37	130 ^d–h^	132 ^e^	2	1.3 ^ci^	1.34 ^c–h^	0.04
30	SAGL 22 -115	7.12 ^g–t^	7.23 ^d–k^	0.11	4.4 ^i–n^	4.46 ^V–IX^	0.06	28.12 ^v–II^	37.45 ^x–II^	9.33	5.21 ^w–II^	9.54 ^w–x^	4.33	169 ^VI–IX^	187 ^V^	18	1.76 ^sw^	1.86 ^s–t^	0.1
31	SAGL-152403	6.32 ^c–t^	6.58 ^a–k^	0.26	2.45 ^a–n^	3.91 ^l–q^	1.46	20.12 ^h–s^	24.12 ^g–r^	4	3.62 ^e–h^	7.64 ^s–u^	4.02	157 ^u–III^	162 ^u^	5	1.67 ^rv^	1.73 ^q–s^	0.06
32	SAGL-152254	5.12 ^a–f^	5.37 ^a–g^	0.25	3.29 ^a–n^	4.25 ^w–IV^	0.96	27.45 ^u–II^	30.25 ^o–x^	2.8	4.52 ^o–s^	6.52 ^o–r^	2	148 ^o–u^	152 ^o^	4	1.3 ^cl^	1.37 ^d–k^	0.07
33	SAGL-162370	5.34 ^a–j^	5.64 ^a–i^	0.3	1.26 ^ab^	4.02 ^n–t^	2.76	25.42 ^p–I^	28.62 ^n–w^	3.2	4.85 ^t–v^	5.61 ^h–m^	0.76	140 ^j–o^	143 ^l^	3	1.62 ^rt^	1.64 ^m–q^	0.02
34	SAGL-152210	7.32 ^i–t^	7.41 ^e–k^	0.09	4.12 ^f–n^	4.27 ^x–V^	0.15	25.42 ^o–I^	35.23 ^u–II^	9.81	5.43 ^z–III^	9.64 ^w–x^	4.21	180 ^XI^	199 ^VIII^	19	1.8 ^ux^	1.98 ^t–u^	0.18
35	SAGL-152273	5.42 ^a–k^	5.65 ^a–i^	0.23	2.34 ^a–k^	4.05 ^o–v^	1.71	15.42 ^c–k^	17.52 ^a–k^	2.1	3.76 ^f–j^	5.64 ^i–m^	1.88	150 ^p–x^	153 ^op^	3	1.32 ^cl^	1.37 ^d–k^	0.05
36	SAGL-152216	4.12 ^ab^	4.26 ^a^	0.14	1.62 ^a–e^	4.04 ^o–u^	2.42	9.85 ^a–d^	13.25 ^a–f^	3.4	4.56 ^p–s^	6.34 ^o–q^	1.78	129 ^c–h^	133 ^ef^	4	1.21 ^be^	1.27 ^bcde^	0.06
37	RVSSG-64	6.37 ^c–t^	6.64 ^a–k^	0.27	2.42 ^a–n^	3.86 ^j–o^	1.44	15.24 ^b–k^	19.52 ^c–m^	4.28	3.58 ^d–f^	4.85 ^r–s^	1.27	162 ^II–VII^	167 ^wx^	5	1.8 ^ux^	1.85 ^q–s^	0.05
38	SAGL-162265	6.98 ^f–t^	7.25 ^d–k^	0.27	2.62 ^a–n^	3.78 ^h–m^	1.16	8.46 ^a–c^	12.32 ^a–e^	3.86	4.12 ^k–m^	6.34 ^o–q^	2.22	138 ^h–n^	141 ^jk^	3	1.1 ^ab^	1.14 ^ab^	0.04
39	SAGL-152347	6.75 ^a–q^	7.12 ^a–k^	0.37	3.49 ^a–n^	4.17 ^z–III^	0.68	9.51	12.51 ^a–e^	3	4.62 ^q–u^	7.45	2.83	153 ^r–I^	157	4	1.29 ^dl^	1.34	0.05
40	SAGL-162376	5.67 ^a–o^	5.9 ^a–k^	0.23	2.34 ^a–k^	4.1 ^p–x^	1.76	10.23 ^a–d^	18.65 ^b–l^	8.42	3.68 ^e–i^	5.24 ^e–h^	1.56	158 ^v–IV^	165 ^vw^	7	1.31 ^cl^	1.36 ^d–i^	0.05
41	SAGL-152314	5.49 ^a–l^	5.75 ^a–j^	0.26	2.42 ^a–n^	4.31 ^z–VI^	1.89	14.32 ^a–j^	17.56 ^a–k^	3.24	4.37 ^m–q^	5.99 ^m–o^	1.62	135 ^g–l^	138 ^i^	3	1.55 ^nr^	1.59 ^l–q^	0.04
42	SAGL-162375	6.85 ^f–t^	7.12 ^c–k^	0.27	2.58 ^a–n^	3.75 ^g–m^	1.17	13.42 ^a–h^	16.42 ^a–i^	3	3.85 ^h–k^	4.65 ^b–c^	0.8	153 ^r–I^	157 ^st^	4	1.3 ^cj^	1.34 ^c–h^	0.04
43	SAGL-152278	7.68 ^o–t^	7.74 ^f–k^	0.06	4.02 ^f–n^	4.12 ^r–y^	0.1	25.12 ^n–I^	37.68 ^x–II^	12.56	5.02 ^v–y^	12.34 ^I^	7.32	165 ^III–VIII^	180 ^III^	15	2.05 ^II^	2.19 ^vw^	0.14
44	SAGL-152242	5.62 ^a–n^	5.89 ^a–k^	0.27	3.25 ^a–n^	4.24 ^v– IV^	0.99	15.32 ^c–k^	20.31 ^e–n^	4.99	3.24 ^b–d^	5.65 ^j^	2.41	138 ^h–n^	142 ^kl^	4	1.29 ^ch^	1.34 ^c–h^	0.05
45	SAGL-152238	8.12 ^s–t^	8.23 ^j–k^	0.11	3.95 ^f–n^	4.6 ^VIII–XI^	0.65	19.52 ^f–p^	27.9 ^m–v^	8.38	5.34 ^y–II^	9.62 ^w–x^	4.28	159 ^x–IV^	175 ^I^	16	1.79 ^tw^	1.98 ^t–u^	0.19
46	SAGL-162390	7.98 ^r–t^	8.21 ^j–k^	0.23	4.23 ^g–n^	4.35 ^I–VI^	0.12	27.32 ^t–I^	42.32 ^I–II^	15	5.67 ^III–IV^	10.24 ^v^	4.57	179 ^X–XI^	192 ^VII^	13	2.04 ^I–II^	2.15 ^v–w^	0.11
47	RVSSG-69	6.37 ^c–t^	6.92 ^b–k^	0.55	3.52 ^a–n^	4.21 ^u–III^	0.69	25.31 ^n–I^	29.61 ^o–x^	4.3	4.28 ^m–o^	7.45 ^s–t^	3.17	133 ^g–k^	135 ^gh^	2	1.32 ^cl^	1.37 ^d–k^	0.05
48	SAGL-152256	5.45 ^a–l^	5.65 ^a–i^	0.2	2.45 ^a–n^	4.2 ^t–III^	1.75	21.42 ^k–v^	28.02 ^m–w^	6.6	4.64 ^r–u^	6.59 ^p–r^	1.95	117 ^a^	121 ^b^	4	1.25 ^bg^	1.29 ^b–g^	0.04
49	SAGL-152208	7.85 ^p–t^	7.9 ^h–k^	0.05	3.35 ^a–n^	4.58 ^VII–X^	1.23	26.31 ^q–Iv^	35.42 ^u–II^	9.11	5.62 ^III–IV^	9.87 ^x^	4.25	174 ^VIII–IX^	185 ^IV^	11	1.26 ^bg^	1.38 ^e–k^	0.12
50	SAGL-152303	6.52 ^d–t^	6.68 ^a–k^	0.16	3.12 ^a–n^	4.31 ^z–VI^	1.19	28.45 ^w–II^	31.25 ^q–z^	2.8	3.42 ^c–e^	5.42 ^o–q^	2	141 ^k–p^	146 ^m^	5	1.4 ^ho^	1.44 ^h–l^	0.04
51	SAGL-152404	4.62 ^a–e^	4.78 ^a–d^	0.16	2.45 ^a–n^	4.3 ^y–VI^	1.85	15.62 ^c–k^	19.85 ^e–m^	4.23	3.65 ^e–h^	6.57 ^p–r^	2.92	140 ^j–o^	145 ^m^	5	1.29 ^ch^	1.35 ^c–h^	0.06
52	SAGL-152236	5.47 ^a–l^	5.75 ^a–j^	0.28	2.12 ^a–h^	3.67 ^f–k^	1.55	29.65 ^w–II^	32.02 ^r–z^	2.37	4.26 ^l–o^	6.95 ^r–s^	2.69	146 ^n–s^	150 ^n^	4	1.61 ^qs^	1.65 ^p–q^	0.04
53	SAGL-152252	6.85 ^f–t^	6.95 ^b–k^	0.1	4.79 ^m–n^	4.82 ^XI–XII^	0.03	30.25 ^y–II^	39.75 ^y–II^	9.5	5.16 ^w–z^	11.25 ^z^	6.09	160 ^y–V^	178 ^II^	18	1.87 ^w–I^	2.03 ^u–v^	0.16
54	SAGL-152349	5.32 ^a–i^	5.46 ^a–h^	0.14	1.52 ^a–d^	3.25 ^cd^	1.73	18.52 ^e–n^	20.32 ^e–n^	1.8	4.32 ^m–p^	6.35 ^o–q^	2.03	152 ^q–z^	156 ^rs^	4	1.65 ^ru^	1.69 ^p–r^	0.04
55	SAGL-162389	5.02 ^c–t^	5.35 ^a–k^	0.33	1.52 ^a–n^	4.12 ^s–t^	2.6	14.26 ^a–e^	18.65 ^a–g^	4.39	4.62 ^p–t^	7.54 ^s–u^	2.92	153 ^r–I^	158 ^st^	5	1.4 ^ho^	1.43 ^e–k^	0.03
56	SAGL-162371	5.98 ^a–r^	6.12 ^a–k^	0.14	2.42 ^a–l^	3.65 ^f–i^	1.23	25.42 ^p–I^	29.56 ^o–x^	4.14	4.25 ^l–o^	6.58 ^p–r^	2.33	160 ^y–VI^	165 ^V^	5	1.31 ^cl^	1.38 ^e–k^	0.07
57	SAGL-152342	6.36 ^c–t^	6.45 ^a–k^	0.09	2.2 ^a–h^	3.24 ^cd^	1.04	28.45 ^w–II^	31.02 ^p–y^	2.57	4.98 ^v–x^	7.54 ^s–u^	2.56	121 ^a–e^	125 ^d^	4	1.92 ^w–II^	1.98 ^t–u^	0.06
58	SAGL-152334	7.24 ^h–t^	7.54 ^e–k^	0.3	2.32 ^a–k^	4.25 ^x–VI^	1.93	26.42 ^r–I^	29.65 ^o–x^	3.23	4.62 ^q–u^	6.52 ^o–r^	1.9	166 ^IV–IX^	170 ^y^	4	1.31 ^cl^	1.35 ^c–h^	0.04
59	RVSSG-75	4.57 ^a–d^	4.69 ^abc^	0.12	3.03 ^a–n^	3.52 ^e–f^	0.49	23.62 ^m–y^	27.42 ^m–u^	3.8	3.52 ^d–f^	4.97 ^c–e^	1.45	129 ^bg^	134 ^fg^	5	1.53 ^mr^	1.58 ^l–o^	0.05
60	JG-14	6.39 ^c–t^	6.5 ^a–k^	0.11	2.32 ^a–i^	4.23 ^u–III^	1.91	12.32 ^a–f^	16.52 ^a–j^	4.2	4.62 ^q–u^	6.57 ^p–r^	1.95	120 ^abc^	123 ^c^	3	1.35 ^fi^	1.37 ^d–k^	0.02
61	JG-11	6.42 ^d–t^	6.49 ^a–k^	0.07	4.55 ^l–n^	4.67 ^IX–XII^	0.12	25.23 ^n–I^	35.42 ^g–q^	10.19	5.26 ^w–II^	10.25 ^y^	4.99	175 ^IX–XI^	187 ^V^	12	1.22 ^cf^	1.29 ^b–g^	0.07
62	NBeG-47	4.62 ^a–d^	4.87 ^a–d^	0.25	1.34 ^abc^	3.25 ^f–k^	1.91	8.45 ^a–^	10.23 ^abc^	1.78	3.24 ^b–d^	6.54 ^p–r^	3.3	115 ^a^	118 ^a^	3	1.83 ^vy^	1.86 ^s–t^	0.03
63	JGG-1	5.85 ^a–q^	5.9 ^a–k^	0.05	2.42 ^a–n^	2.89 ^ab^	0.47	7.45 ^a^	11.65 ^a–e^	4.2	3.98 ^j–l^	4.25 ^a^	0.27	145 ^m–r^	152 ^o^	7	1.38 ^fn^	1.41 ^g–k^	0.03
64	RVG-205	6.12 ^a–s^	6.34 ^a–k^	0.22	2.54 ^a–n^	3.21 ^cd^	0.67	9.85 ^a–d^	12.34 ^a–e^	2.49	3.75 ^f–j^	6.54 ^p–r^	2.79	132 ^f–j^	138 ^i^	6	1.3 ^ch^	1.34 ^c–h^	0.04
65	RVG-201	6.46 ^d–t^	6.54 ^a–k^	0.08	2.64 ^a–n^	4.51 ^bc^	1.87	20.13 ^h–s^	25.32 ^k–s^	5.19	4.58 ^p–t^	6.34 ^q–s^	1.76	162 ^I–VII^	165 ^v^	3	1.31 ^cl^	1.36 ^c–i^	0.05
66	VISHAL	7.42 ^k–t^	7.58 ^e–k^	0.16	3.24 ^a–n^	4.12 ^q–y^	0.88	31.42 ^I–II^	34.12 ^t–I^	2.7	4.76 ^s–v^	5.87 ^l–n^	1.11	130 ^d–h^	133 ^ef^	3	1.65 ^ru^	1.68 ^o–q^	0.03
67	JG-63	6.21 ^a–s^	6.34 ^a–k^	0.13	3.62 ^d–n^	4.35 ^I–VI^	0.73	16.42 ^d–l^	19.65 ^d–m^	3.23	3.95 ^i–k^	4.32 ^a^	0.37	155 ^s–II^	158 ^t^	3	1.29 ^ch^	1.34 ^c–h^	0.05
68	RVSSG-85	7.61 ^m–t^	7.75 ^g–k^	0.14	4.51 ^j–n^	4.77 ^X–XII^	0.26	30.54 ^z–II^	44.25 ^II^	13.71	5.46 ^I–III^	9.65 ^w–x^	4.19	168 ^V–IX^	180 ^III^	12	2.06 ^II^	2.18 ^v–w^	0.12
69	RVG-210	6.62 ^e–t^	6.72 ^a–k^	0.1	2.42 ^a–n^	3.6 ^e–i^	1.18	19.65 ^f–q^	22.32 ^f–o^	2.67	5.02 ^II–IV^	7.62 ^s–u^	2.6	139 ^i–o^	142 ^kl^	3	1.45 ^kq^	1.49 ^i–m^	0.04
70	SAGL-161032	4.65 ^a–e^	4.85 ^a–d^	0.2	3.06 ^a–n^	3.51 ^e–f^	0.45	25.42 ^p–I^	29.62 ^o–x^	4.2	4.98 ^v–w^	5.24 ^e–h^	0.26	160 ^y–VI^	163 ^u^	3	1.6 ^qs^	1.64 ^m–q^	0.04
71	SAGL-163603	6.32 ^c–t^	6.52 ^a–k^	0.2	3.24 ^a–n^	4.21 ^t–III^	0.97	20.12 ^h–s^	24.62 ^i–s^	4.5	3.62 ^e–h^	5.45 ^g–l^	1.83	119 ^ab^	124 ^cd^	5	2.08 ^II^	2.13 ^u–w^	0.05
72	SAGL-161018	5.34 ^a–j^	5.49 ^a–h^	0.15	1.25 ^a^	3.8 ^i–n^	2.55	27.45 ^u–II^	30.12 ^o–x^	2.67	4.56 ^p–s^	5.75 ^k–n^	1.19	131 ^f–j^	135 ^gh^	4	1.31 ^cl^	1.37 ^d–k^	0.06
73	SAGL-163008	6.34 ^c–t^	6.57 ^a–k^	0.23	2.42 ^a–n^	3.6 ^e–i^	1.18	25.43 ^p–I^	29.85 ^o–x^	4.42	3.28 ^c–d^	4.35 ^t–u^	1.07	120 ^abcd^	123 ^c^	3	1.25 ^bg^	1.29 ^bcde^	0.04
74	SAGL-161001	5.12 ^a–g^	5.32 ^a–f^	0.2	2.65 ^a–n^	3.54 ^e–g^	0.89	26.34 ^r–I^	31.26 ^q–z^	4.92	4.39 ^n–r^	6.58 ^p–r^	2.19	145 ^m–r^	149 ^n^	4	1.4 ^ho^	1.45 ^h–l^	0.05
75	RVSSG-68	5.75 ^a–o^	5.86 ^a–j^	0.11	2.75 ^a–n^	3.41 ^de^	0.66	30.12 ^x–II^	33.25 ^p–z^	3.13	3.26 ^b–d^	5.85 ^s–t^	2.59	149 ^m–r^	154	5	1.61 ^qs^	1.65 ^o–q^	0.04
76	JG-315	7.34 ^j–t^	7.39 ^b–k^	0.05	4.52 ^k–n^	4.78 ^X–XII^	0.26	27.42 ^j–u^	40.21 ^z–II^	12.79	5.98 ^V–VI^	11.24 ^z^	5.26	175 ^VIII–IX^	190 ^VI^	15	2.01 ^z–II^	2.2 ^w^	0.19
77	JG-74	6.32 ^b–t^	6.64 ^a–k^	0.32	2.32 ^a–j^	3.72 ^f–l^	1.4	30.12 ^w–II^	32.62 ^s–z^	2.5	3.62 ^e–h^	4.42 ^ab^	0.8	170 ^VII–X^	172 ^z^	2	1.62 ^rt^	1.64 ^n–q^	0.02
78	ICC-4958	8.02 ^r–t^	8.06 ^i–k^	0.04	4.81 ^n^	4.89 ^XII^	0.08	28.45 ^i–t^	42.32 ^I–II^	13.87	6.01 ^V–VI^	13.52 ^II^	7.51	174 ^VIII–XI^	192 ^VII^	18	1.97 ^x–II^	2.15 ^v–w^	0.18
	Mean	6.22	6.41	0.19	3.05	4.08	1.03	20.96	26.48	5.52	4.40	6.90	2.50	148.54	155.62	7.08	1.52	1.59	0.07
	Max.	8.32	8.41	0.55	4.81	4.89	2.76	35.02	44.46	17.07	6.12	13.52	7.51	180.00	199.00	19.00	2.09	2.20	0.19
	Min.	4.02	4.26	0.04	1.25	2.89	0.03	7.42	9.23	1.72	2.65	4.25	0.26	115.00	118.00	2.00	0.98	1.02	0.01
	SD	1.029	0.995		0.936	0.426		7.089	9.187		0.838	2.058		16.72	19.75		0.28	0.31	
	SE	0.07	0.07		0.06	0.04		0.50	0.62		0.06	0.13		1.09	1.30		0.03	0.04	

**Table 5:** Correlation analysis between drought and normal conditions for non-enzymatic biochemical parameters.

	MDA N	MDA D	Sugar N	Sugar D	Phenol N	Phenol D	Proline N	Proline D	Protein N	Protein D	Chla N	Chla D	Chlb N	Chlb D
MDA N	1.0000													
MDA D	0.9961 **	1.0000												
Sugar N	0.6263 **	0.6137 **	1.0000											
Sugar D	0.5140 **	0.5126 **	0.6619 **	1.0000										
Phenol N	0.3604 **	0.3442 **	0.3366 **	0.2816 **	1.0000									
Phenol D	0.5387 **	0.5149 **	0.5464 **	0.4559 **	0.9296 **	1.0000								
Proline N	0.5668 **	0.5392 **	0.5034 **	0.4195 **	0.2531 **	0.4675 **	1.0000							
Proline D	0.5982 **	0.5707 **	0.6122 **	0.5305 **	0.3638 **	0.6055 **	0.7749 **	1.0000						
Protein N	0.0573	0.0438	0.1787	0.2124	0.1897	0.1079	0.2643 *	0.2380 *	1.0000					
Protein D	0.2216	0.1930	0.2824 *	0.3891	0.3247	0.2582	0.3948 **	0.4107 **	0.9411 **	1.0000				
Chl_a_ N	0.6091 **	0.4771 **	0.4382 **	0.2400 **	0.4660 **	0.6051 **	0.4133 **	0.5766 **	0.3378	0.2111	1.0000			
Chl_a_ D	0.6002 **	0.5209 **	0.4986 **	0.3011 **	0.4782 **	0.6397 **	0.4767 **	0.6429 **	0.3850	0.2831 *	0.7803 **	1.0000		
Chl_b_ N	0.5009 **	0.4489 **	0.4531 **	0.6027 **	0.3907 **	0.4184 **	0.2659 **	0.4189 **	0.1484	0.2993 **	0.5782 **	0.5469 **	1.0000	
Chl_b_ D	0.5332 **	0.4714 **	0.4219 **	0.6327 **	0.4424 **	0.4670 **	0.3178 **	0.4487 **	0.2282 *	0.3853 **	0.6139 **	0.6099 **	0.8714 **	1.0000

N—normal condition D—drought condition Significant level 0.05 * and 0.01 **.

**Table 6:** Correlation analysis between drought and normal conditions for antioxidant enzyme activity.

	DPPH N	DPPH D	H_2_O_2_N	H_2_O_2_D	CAT N	CAT D	Ascorbate n	Ascorbate d	SOD N	SOD D	POX N	POX D
DPPH N	1.0000											
DPPH D	0.9961 **	1.0000										
H_2_O_2_ N	0.6263 **	0.6137 **	1.0000									
H_2_O_2_ D	0.5140 **	0.5126 **	0.6619 **	1.0000								
CAT N	0.3604 **	0.3442 **	0.3366 **	0.2816 **	1.0000							
CAT D	0.5387 **	0.5149 **	0.5464 **	0.4559 **	0.9296 **	1.0000						
Ascorbate N	0.5668 **	0.5392 **	0.5034 **	0.4195 **	0.2531 **	0.4675 **	1.0000					
Ascorbate D	0.5982 **	0.5707 **	0.6122 **	0.5305 **	0.3638 **	0.6055 **	0.7749 **	1.0000				
SOD N	0.4824 **	0.4689 **	0.5269 **	0.4545 **	0.3514 ****	0.5164 **	0.3754 **	0.4824 **	1.0000			
SOD D	0.5629 **	0.5437 **	0.6272 **	0.5121 **	0.3804 **	0.5791 **	0.4702 **	0.5910 **	0.9798 **	1.0000		
POX N	0.4996 **	0.4771 **	0.4382 **	0.2400 *	0.4660 **	0.6051 **	0.4133 **	0.5766 **	0.3378 **	0.4231 **	1.0000	
POX D	0.5460 **	0.5209 **	0.4986 **	0.3011 **	0.4782 **	0.6397 **	0.4767 **	0.6429 **	0.3850 **	0.4819 **	0.9927 **	1.0000

N—normal conditions; D—drought conditions. Significance levels of 0.05 * and 0.01 **.

**Table 7:** (a) PCA, eigenvalue, variability, and cumulative % for non-enzymatic biochemical parameters. (b) PCA, eigenvalue, variability, and cumulative % for enzymatic biochemical parameters or antioxidant activity.

Traits	Principal Component (PC)	Eigenvalue	Variability (%)	Cumulative (%)
(a)				
MDA (Normal)	PC1	6.88	49.16	49.16
MDA (Drought)	PC2	1.90	13.57	62.73
Sugar (Normal)	PC3	1.30	9.27	72
Sugar (Drought)	PC4	1.12	8.01	80.01
Phenol (Normal)	PC5	0.83	5.93	85.94
Phenol (Drought)	PC6	0.70	5.02	90.96
Proline (Normal)	PC7	0.49	3.47	94.43
Proline (Drought)	PC8	0.27	1.96	96.38
Protein (Normal)	PC9	0.14	1.02	97.41
Protein (Drought)	PC10	0.12	0.83	98.23
Chl_a_ (Normal)	PC11	0.09	0.64	98.87
Chl_a_ (Drought)	PC12	0.08	0.57	99.44
Chl_b_ (Normal)	PC13	0.07	0.49	99.93
Chl_b_ (Drought)	PC14	0.01	0.06	100
(b)				
DPPH N	PC1	6.77	56.41	56.41
DPPH D	PC2	1.42	11.83	68.24
H2O2 N	PC3	1.12	9.33	77.57
H2O2 D	PC4	0.84	7.02	84.59
CAT N	PC5	0.73	6.11	90.7
CAT D	PC6	0.59	4.95	95.65
Ascorbate n	PC7	0.31	2.54	98.19
Ascorbate d	PC8	0.18	1.52	99.71
SOD N	PC9	0.02	0.17	99.88
SOD D	PC10	0.01	0.08	99.96
POX N	PC11	0.00	0.03	99.99
POX D	PC12	0.00	0.01	100

**Table 8:** Details of experimental material with their parentage.

S. No.	Genotype	Pedigree	S. No.	Genotype	Pedigree
1	ICCV-201111	JNKVV, Jabalpur	40	SAGL-162376	JSC 52 × RSG 888
2	JG-36	JG 12 × JG 16	41	SAGL-152314	KAK 2 × VISHAL
3	GCP-101	JNKVV, Jabalpur	42	SAGL-162375	JAKI 9218 × JSC 52
4	ICCV-20 1105	JNKVV, Jabalpur	43	SAGL-152278	JSC 37 × JSC 36
5	ICCV-201209	JNKVV, Jabalpur	44	SAGL-152242	PG 9425-9 × BG 1108
6	ICCV-201113	JNKVV, Jabalpur	45	SAGL-152238	PG 9425-9 × IPC 9494
7	ICCV-201118	JNKVV, Jabalpur	46	SAGL-162390	JSC 37 × JSC 36
8	ICCV-201212	JNKVV, Jabalpur	47	RVSSG-69	RAK, Sehore
9	ICCV-201217	JNKVV, Jabalpur	48	SAGL-152256	JSC 19 × KAK 2
10	ICCV-201102	JNKVV, Jabalpur	49	SAGL-152208	BG 362 × IPC 9494
11	ICCV-201218	JNKVV, Jabalpur	50	SAGL-152303	JSC 19 × BGD 112
12	ICCV-201108	JNKVV, Jabalpur	51	SAGL-152404	RAK, Sehore
13	CHAFFA	JNKVV, Jabalpur	52	SAGL-152236	KAK 2 × BG 362
14	JG-24	(JG 74 × ICC 4958)-21	53	SAGL-152252	ICC 4958 × BG 1108
15	ICCV-201114	JNKVV, Jabalpur	54	SAGL-152349	KAK 2 × PHULE G5
16	ICCV-201107	JNKVV, Jabalpur	55	SAGL-162389	ICC4812 × JAKI 9218
17	SAGL 22-101	KAK 2 × BG 362	56	SAGL-162371	JSC 52 ×JG 130
18	SAGL 22-102	JG -6 × RVSSG 2	57	SAGL-152342	KAK 2 × JSC 19
19	SAGL 22-103	JG 130 × FG 703	58	SAGL-152334	PG 9425-9 × IPC 9494
20	SAGL 22-104	JSC 33 × JG 11	59	RVSSG-75	RAK, Sehore
21	SAGL 22-105	JAKI 9218 × BGD 112	60	JG-14	(GW5/7 × P326) × ICCL83149
22	SAGL 22-106	RVG 204 × JSC 37	61	JG-11	(Phule G-5 × Narsinghpur bold) × ICCC37
23	SAGL 22-107	RVG 202 × JG 11	62	NBeG-47	RAK, Sehore
24	SAGL 22-108	JAKI 9218 × RSG 888	63	JGG-1	RAK, Sehore
25	SAGL 22-109	JG 11 × JSC 37	64	RVG-205	RAK, Sehore
26	SAGL 22-111	JG 130 × JSC 37	65	RVG-201	Phule G5 × Bheema
27	SAGL 22-112	RVSSG 74 × GBM 2	66	VISHAL	RAK, Sehore
28	SAGL 22-113	JSC 38 × IPCK 1078	67	JG-63	Single Plant selection from JG 62
29	SAGL 22-114	RVSSG 74 × ICC4958	68	RVSSG-85	RAK, Sehore
30	SAGL 22 -115	SG 9200 × BG 362	69	RVG-210	RAK, Sehore
31	SAGL-152403	RAK, Sehore	70	SAGL-161032	RAK, Sehore
32	SAGL-152254	BG 362 × ICC 506	71	SAGL-163603	RAK, Sehore
33	SAGL-162370	PG 9425-9 × BG 2064	72	SAGL-161018	JG 130 × BGD 112
34	SAGL-152210	IPC 9494 × ICC 506	73	SAGL-163008	ICC 4812 × RSG 888
35	SAGL-152273	KAK 2 × IPC 9494	74	SAGL-161001	JSC 52 × BGD 112
36	SAGL-152216	JG 16 × VIJAY	75	RVSSG-68	RAK, Sehore
37	RVSSG-64	RAK, Sehore	76	JG-315	JGM 1 × ICC 4929
38	SAGL-162265	BG 362 × JSC 19	77	JG-74	A composite from genetic stock
39	SAGL-152347	KAK 2 × JSC 19	78	ICC-4958	JGC 4958

(Source—genotypes acquired from AICRP on Chickpea, RAK College of Agriculture, Sehore, RVSKVV, Gwalior, M.P., India and AICRP on Chickpea College of Agriculture, JNKVV, Jabalpur, M.P., India).

## Data Availability

The original contributions presented in the study are included in the article, further inquiries can be directed to the corresponding author.

## References

[1] Azizian A., Fotovat R., Bahramnejad B., Kanouni H. (2024). Drought effects on shoot traits and introduction of new indices in chickpea to identify drought tolerant and susceptible genotypes. Genet. Resour. Crop Evol..

[2] Tiwari P.N., Tiwari S., Sapre S., Babbar A., Tripathi N., Tiwari S., Tripathi M.K. (2023). Prioritization of Microsatellite Markers Linked with Drought Tolerance Associated Traits in Chickpea (*Cicer arietinum* L.). Legum. Res..

[3] Shafiq M., Arif M., Akhtar N., Yousaf M., Ghaffar A. (2021). Exogenous application of growth promoters can improve the chickpea productivity under terminal heat stress conditions by modulating the antioxidant enzyme system. Pak. J. Agri. Sci..

[4] Cutforth H.W., McGinn S.M., McPhee K.E., Miller P.R. (2007). Adaptation of pulse crops to the changing climate of the northern Great Plains. Agron. J..

[5] Bohra A., Tiwari A., Kaur P., Ganie S.A., Raza A., Roorkiwal M., Mir R.R., Fernie A.R., Smýkal P., Varshney R.K. (2022). The Key to the Future Lies in the Past: Insights from Grain Legume Domestication and Improvement Should Inform Future Breeding Strategies. Plant Cell Physiol..

[6] Gaur P.M., Samineni S., Thudi M., Tripathi S., Sajja S.B., Jayalakshmi V., Mannur D.M., Vijayakumar A.G., Ganga Rao N.V., Ojiewo C. (2019). Integrated breeding approaches for improving drought and heat adaptation in chickpea (*Cicer arietinum* L.). Plant Breed..

[7] Wallace T.C., Murray R., Zelman K.M. (2016). The Nutritional Value and Health Benefits of Chickpeas and Hummus. Nutrients.

[8] Kushwah A., Bhatia D., Singh G., Singh I., Bindra S., Vij S., Singh S. (2021). Phenotypic evaluation of genetic variability and selection of yield contributing traits in chickpea recombinant inbred line population under high temperature stress. Physiol. Mol. Biol. Plants.

[9] (2021). FAOSTAT. https://www.fao.org/statistics/en.

[10] Korbu L., Tafes B., Kassa G., Mola T., Fikre A. (2020). Unlocking the genetic potential of chickpea through improved crop management practices in Ethiopia. A review. Agron. Sustain. Dev..

[11] Dotaniya C.K., Lakaria B.L., Sharma Y., Meena B.P., Aher S.B., Shirale A.O., Pandurang P.G., Dotaniya M.L., Biswas A.K., Patra A.K. (2022). Performance of chickpea (*Cicer arietinum* L.) in maize-chickpea sequence under various integrated nutrient modules in a Vertisol of Central India. PLoS ONE.

[12] Talebi R., Ensafi M.H., Baghebani N., Karami E., Mohammadi K. (2013). Physiological responses of chickpea (*Cicer arietinum* L.) genotypes to drought stres. Environ. Exp. Biol..

[13] Kaloki P., Luo Q., Trethowan R., Tan D.K. (2019). Can the development of drought tolerant ideotype sustain Australian chickpea yield?. Int. J. Biometeorol..

[14] Maqbool M.A., Aslam M., Ali H. (2017). Breeding for improved drought tolerance in Chickpea (*Cicer arietinum* L.). Plant Breed..

[15] Tiwari P.N., Tiwari S., Sapre S., Babbar A., Tripathi N., Tiwari S., Tripathi M.K. (2023). Screening and Selection of Drought-Tolerant High-Yielding Chickpea Genotypes Based on Physio-Biochemical Selection Indices and Yield Trials. Life.

[16] Sharma V., Kaur J., Singh S., Singh I., Kaur S., Johal N. (2017). Physiological and biochemical adaptation of chickpea (*Cicer arietinum* L.) genotypes under moisture stress. J. Food Legumes.

[17] Seifikalhor M., Niknam V., Aliniaeifard S., Didaran F., Tsaniklidis G., Fanourakis D., Teymoorzadeh M., Mousavi S.H., Bosacchi M., Li T. (2022). The regulatory role of γ-aminobutyric acid in chickpea plants depends on drought tolerance and water scarcity level. Sci. Rep..

[18] Purushothaman R., Krishnamurthy L., Upadhyaya H.D., Vadez V., Varshney R.K. (2016). Shoot traits and their relevance in terminal drought tolerance of chickpea (*Cicer arietinum* L.). Field Crop Res..

[19] Mafakheri A., Siosemardeh A., Bahramnejad B., Struik P.C., Sohrabi Y. (2010). Effect of drought stress on yield, proline and chlorophyll contents in three chickpea cultivars. Aust. J. Crop. Sci..

[20] Farooq M., Hussain M., Nawaz A., Lee D.J., Alghamdi S.S., Siddique K.H. (2017). Seed priming improves chilling tolerance in chickpea by modulating germination metabolism, trehalose accumulation and carbon assimilation. Plant PhysiolBiochem..

[21] Farooq M., Ullah A., Lee D.J., Alghamdi S.S., Siddique K.H. (2018). Desi chickpea genotypes tolerate drought stress better than kabuli types by modulating germination metabolism, trehalose accumulation and carbon assimilation. Plant PhysiolBiochem..

[22] Kumar A., Kumar A., Ranjan R., Kumar S., Rajani K., Singh P. (2019). Principal component analysis of Agro-morpho- genetic traits in Desi chickpea (*Cicer arietinum* L.). Int. J. Chem..

[23] Pappula-Reddy S.-P., Kumar S., Pang J., Chellapilla B., Pal M., Millar A.H., Siddique K.H. (2024). High-throughput phenotyping for terminal drought stress in chickpea (*Cicer arietinum* L.). Plant Stress.

[24] Yadav R.K., Tripathi M.K., Tiwari S., Tripathi N., Asati R., Patel V., Sikarwar R.S., Payasi D.K. (2023). Breeding and Genomic Approaches towards Development of Fusarium Wilt Resistance in Chickpea. Life.

[25] Kabbadj A., Makoudi B., Mouradi M., Pauly N., Frendo P., Ghoulam C. (2017). Physiological and biochemical responses involved in water deficit tolerance of nitrogen-fixing *Vicia faba*. PLoS ONE.

[26] Zlatev Z.S. (2013). Drought-induced changes and recovery of photosynthesis in two bean cultivars (*Phaseolus vulgaris* L.). Emirates J. Food Agric..

[27] Noctor G., Mhamdi A., Foyer C.H. (2014). Update on the Physiology of Reactive Oxygen Metabolism during Drought the Roles of Reactive Oxygen Metabolism in Drought: Not So Cut and Dried. Plant Physiol..

[28] You J., Chan Z. (2015). ROS Regulation During Abiotic Stress Responses in Crop Plants. Front. Plant Sci..

[29] Rizvi A.H., Dwivedi V.K., Sairam R.K., Yadav S.S., Bharadwaj C., Sarker A., Alam A. (2014). Physiological Studies on Moisture Stress Tolerance in Chickpea (*Cicer Arietinum* L.) Genotypes. Int. J. Sci. Res. Agric. Sci..

[30] Mishra N., Tripathi M.K., Tiwari S., Tripathi N., Gupta N., Sharma A. (2021). Morphological and Physiological Performance of Indian Soybean [*Glycine max* (L.) Merrill] Genotypes in Respect to Drought. Legume Res..

[31] Karimizadeh R., Mohammadi M. (2011). Association of canopy temperature depression with yield of durum wheat genotypes under supplementary irrigated and rainfed conditions. Aust. J. Crop Sci..

[32] Shakeel A.A., Xiao-Yu X., Long-Chang W., Muhammad F.S., Chen M., Wang L. (2011). Morphological, physiological and biochemical responses of plants to drought stress. Afr. J. Agric. Res..

[33] Rahmatollah K., Kavoos K., Farzad K., Peyman S. (2021). Analysis of Screening Tools for Drought Tolerance in Chickpea (*Cicer arietinum* L.) Genotypes. Agric. Sci. Digest..

[34] Kohakade S.N., Kute N.S., Totre A.S., Kulwal P.L. (2023). Screening of chickpea germplasm lines for drought tolerance based on Relative Water Content (RWC) and Membrane Stability Index (MSI) under rainfed condition. Pharma Innov. J..

[35] Shah T.M., Imran M., Atta B.M., Ashraf M.Y., Hameed A., Waqar I., Shafiq M., Hussain K., Naveed M., Aslam M. (2020). Selection and screening of drought tolerant high yielding chickpea genotypes based on physio-biochemical indices and multi-environmental yield trials. BMC Plant Biol..

[36] Gechev T., Petrov V. (2020). Reactive Oxygen Species and Abiotic Stress in Plants. Int. J. Mol. Sci..

[37] Kumari P., Rastogi A., Yadav S. (2020). Effects of Heat stress and molecular mitigation approaches in orphan legume, Chickpea. Mol. Biol. Rep..

[38] Tuteja N., Tiburcio A.F., Gill S.S., Tuteja R. (2012). Improving Crop Resistance to Abiotic Stress.

[39] Kaur N., Kaur J., Grewal S.K., Singh I. (2019). Effect of Heat Stress on Antioxidative Defense System and Its Amelioration by Heat Acclimation and Salicylic Acid Pre-Treatments in Three Pigeonpea Genotypes. Indian J. Agric. Biochem..

[40] Hasanuzzaman M., Bhuyan M.B., Zulfiqar F., Raza A., Mohsin S.M., Al Mahmud J., Fujita M., Fotopoulos V. (2020). Reactive Oxygen Species and Antioxidant Defense in Plants under Abiotic Stress: Revisiting the Crucial Role of a Universal Defense Regulator. Antioxidants.

[41] Jameel S., Hameed A., Shah T.M. (2021). Investigation of Distinctive Morpho-Physio and Biochemical Alterations in Desi Chickpea at Seedling Stage Under Irrigation, Heat, and Combined Stress. Front. Plant Sci..

[42] Sharma A., Tripathi M.K., Tiwari S., Gupta N., Tripathi N., Mishra N. (2021). Evaluation of Soybean (*Glycine max* L.) Genotypes on the Basis of Biochemical Contents and Anti-oxidant Enzyme Activities. Legume Res..

[43] Kumar T., Bharadwaj C., Tiwari N., Satyavathi C.T., Patil B.S., Sarker A., ALAM A. (2018). Morphological characterization and grouping of chickpea (*Cicer arietinum*) genotypes for drought tolerance. Indian J. Agric. Sci..

[44] Sahu V.K., Tiwari S., Gupta N., Tripathi M.K., Yasin M. (2022). Evaluation of Physiological and Biochemical Contents in Desi and Kabuli Chickpea. Legum. Res..

[45] Zargar S.M., Gupta N., Nazir M., Mahajan R., Malik F.A., Sofi N.R., Shikari A.B., Salgotra R. (2017). Impact of drought on photosynthesis: Molecular perspective. Plant Gene.

[46] Bhagyawant S.S., Gupta N., Shrivastava N. (2015). Biochemical Analysis of Chickpea Accessions vis-a-vis; Zinc, Iron, Total Protein, Proline and Antioxidant Activity. Am. J. Food Sci. Technol..

[47] Furlan A.L., Bianucci E., Giordano W., Castro S., Becker D.F. (2020). Proline metabolic dynamics and implications in drought tolerance of peanut plants. Plant PhysiolBiochem..

[48] Rajput S., Jain S., Tiwari S., Barela A., Chauhan S., Tiwari P.N., Gupta N., Sikarwar R.S., Tripathi N., Tripathi M.K. (2023). Biochemical Characterization of Chickpea (*Cicer arietinum* L.) Genotypes. Plant Cell Biotechnol. Mol. Biol..

[49] de Camargo A.C., Favero B.T., Morzelle M.C., Franchin M., Alvarez-Parrilla E., de la Rosa L.A., Geraldi M.V., Júnior M.R.M., Shahidi F., Schwember A.R. (2019). Is Chickpea a Potential Substitute for Soybean? Phenolic Bioactives and Potential Health Benefits. Int. J. Mol. Sci..

[50] Tiwari P.N., Tiwari S., Sapre S., Tripathi N., Payasi D.K., Singh M., Thakur S., Sharma M., Tiwari S., Tripathi M.K. (2023). Prioritization of Physio-Biochemical Selection Indices and Yield-Attributing Traits toward the Acquisition of Drought Tolerance in Chickpea (*Cicer arietinum* L.). Plants.

[51] Awasthi R., Gaur P., Turner N.C., Vadez V., Siddique K.H.M., Nayyar H. (2017). Effects of individual and combined heat and drought stress during seed filling on the oxidative metabolism and yield of chickpea (*Cicer arietinum*) genotypes differing in heat and drought tolerance. Crop Pasture Sci..

[52] Sadeghi A., Razmjoo J., Karimmojeni H., Baldwin T.C., Mastinu A. (2024). Changes in Secondary Metabolite Production in Response to Salt Stress in *Alcea rosea* L.. Horticulturae.

[53] Sharma I., Ahmad P., Ahmad P. (2014). Catalase: A versatile antioxidant in plants. Oxidative Damage to Plants.

[54] Jan M., Haq T.U., Sattar H., Butt M., Khaliq A., Arif M., Rauf A. (2020). Evaluation and Screening of Promising Drought Tolerant Chickpea (*Cicer arietinum* L.) Genotypes Based on Physiological and Biochemical Attributes Under Drought Conditions. Pak. J. Agric. Res..

[55] Gangwar S., Singh V.P., Tripathi D.K., Chauhan D.K., Prasad S.M., Maurya J.N., Ahmad P., Rasool S. (2014). Plant responses to metal stress: The emerging role of plant growth hormones in toxicity alleviation. Emerging Technologies and Management of Crop Stress Tolerance.

[56] McGill M.R., Jaeschke H., Kaplowitz N., DeLeve L.D. (2013). Oxidant stress, antioxidant defense, and liver injury. Drug-Induced Liver Disease.

[57] Sachdeva S., Bharadwaj C., Patil B.S., Pal M., Roorkiwal M., Varshney R.K. (2022). Agronomic Performance of Chickpea Affected by Drought Stress at Different Growth Stages. Agronomy.

[58] Weatherley P.E. (1950). Studies in the water relations of the cotton plant. I. the field measurement of water deficits in leaves. New Phytol..

[59] Khanna-Chopra R., Selote D.S. (2007). Acclimation to drought stress generates oxidative stress tolerance in drought-resistant than -susceptible wheat cultivar under field conditions. Environ. Exp. Bot..

[60] Arnon D.I. (1949). Copper enzymes in isolated chloroplasts. Polyphenol oxidase in Beta vulgaris. Plant Physiol..

[61] Bates L.S., Waldren R.P., Teare I.D. (1973). Rapid determination of free proline for water-stress studies. Plant Soil.

[62] DuBois M., Gilles K.A., Hamilton J.K., Rebers P.A., Smith F. (1956). Colorimetric method for determination of sugars and related substances. Anal. Chem..

[63] Hodges D.M., DeLong J.M., Forney C.F., Prange R.K. (1999). Improving the thio barbituric acid-reactive-substances assay for estimating lipid peroxidation in plant tissues containing anthocyanin and other interfering compounds. Planta.

[64] Lowry O.H., Rosebrough N.J., Farr A.L., Randall R.J. (1951). Protein measurement with the Folin phenol reagent. J. Biol. Chem..

[65] Swain T., Hillis W.E. (1959). The phenolic constituents of *Prunus domestica*. I.—The quantitative analysis of phenolic constituents. J. Sci. Food Agric..

[66] Sanja S.D., Sheth N.R., Patel N.K., Patel D., Patel B. (2009). Characterization and evaluation of antioxidant activity of Portulaca oleraceae. Int. J. Pharm. Sci..

[67] Alexieva M.S., Bhagwat A.A., Cregan P.B. (2001). Length polymorphisms of simple sequence repeat DNA in soybean. Genetics.

[68] Aebi H. (1984). Catalase in vitro. Methods Enzymol..

[69] Nakano Y., Asada K. (1981). Hydrogen peroxide is scavenged by ascorbate specific peroxidase in spinach chloroplasts. Plant Cell Physiol..

[70] Castillo F.I., Penel I., Greppin H. (1984). Peroxidase release induced by ozone in Sedum album leaves. Plant Physiol..

